# Restoration of the gut-microbiota-liver axis after hepatitis C virus eradication

**DOI:** 10.1016/j.jhepr.2025.101494

**Published:** 2025-06-24

**Authors:** Takako Inoue, Jiro Nakayama, Hiroshi Mori, Masaru Tanaka, Daisuke Nakagawa, Masaya Ohnishi, Yui Funatsu, Kei Moriya, Hideto Kawaratani, Hisayoshi Watanabe, Goki Suda, Yasuteru Kondo, Tatsuya Ide, Satoru Kakizaki, Satoshi Miuma, Atsushi Suetsugu, Kazuhito Kawata, Takao Watanabe, Etsuko Iio, Rie Momoda, Yutaka Suzuki, Akira Sakamaki, Tsunamasa Watanabe, Takehisa Watanabe, Katsuya Nagaoka, Yoichi Hiasa, Shuji Terai, Hitoshi Yoshiji, Atsushi Toyoda, Ken Kurokawa, Yasuhito Tanaka

**Affiliations:** 1 Department of Clinical Laboratory Medicine, Nagoya City University Hospital, Nagoya, Japan; 2 Laboratory of Microbial Technology, Division of Systems Bioengineering, Department of Bioscience and Biotechnology, Faculty of Agriculture, Graduate School, Kyushu University, Fukuoka, Japan; 3 Advanced Genomics Center, National Institute of Genetics, Mishima, Japan; 4 JSR Corporation, Tokyo, Japan; 5 Department of Gastroenterology/Internal Medicine, Gifu University Graduate School of Medicine, Gifu, Japan; 6 Department of Computational Biology and Medical Sciences, Graduate School of Frontier Sciences, The University of Tokyo, Kashiwa, Japan; 7 Department of Gastroenterology and Hepatology, Nara Medical University, Kashihara, Japan; 8 Department of Gastroenterology and Hepatology, Tohoku Central Hospital of the Mutual Aid Association of Public School Teachers, Yamagata, Japan; 9 Department of Gastroenterology and Hepatology, Graduate School of Medicine, Hokkaido University, Sapporo, Japan; 10 Department of Hepatology, Sendai Kousei Hospital, Sendai, Japan; 11 Department of Hepatology, Sendai Tokushukai Hospital, Sendai Japan; 12 Division of Gastroenterology, Kurume University Medical Center, Kurume, Japan; 13 Department of Clinical Research, NHO Takasaki General Medical Center, Takasaki, Japan; 14 Department of Gastroenterology and Hepatology, Nagasaki University Graduate School of Biomedical Sciences, Nagasaki, Japan; 15 Hepatology Division, Department of Internal Medicine II, Hamamatsu University School of Medicine, Hamamatsu, Japan; 16 Department of Gastroenterology and Metabology, Ehime University Graduate School of Medicine, Toon, Japan; 17 Department of Gastroenterology and Hepatology, Faculty of Life Sciences, Kumamoto University, Kumamoto, Japan; 18 Division of Gastroenterology and Hepatology, Graduate School of Medical and Dental Sciences, Niigata University, Niigata, Japan; 19 Division of Gastroenterology and Hepatology, St. Marianna University School of Medicine, Kawasaki, Japan

**Keywords:** chronic hepatitis C (CHC), gut dysbiosis, *Blautia*, metabolomic analysis, transcriptional analysis (RNA-Seq)

## Abstract

**Background & Aims:**

We previously reported altered intestinal environmental features during HCV infection. Here, we aimed to characterize the gut-microbiota-liver axis in patients with chronic hepatitis C after a sustained virological response (SVR).

**Methods:**

A total of 174 patients with HCV infection were enrolled in a cross-sectional study: 95 with chronic hepatitis (CH-HCV group) and 79 with cirrhosis or hepatocellular carcinoma (LC/HCC-HCV group). In addition, 75 post-SVR patients were included (CH-SVR group, n = 29; LC/HCC-SVR group, n = 46), along with 23 healthy individuals. A longitudinal study was subsequently conducted on 49 patients (CH, n = 29; LC/HCC, n = 20) with SVR at 24 and 48 weeks after the end of treatment. RNA sequencing was performed on 65 patients with HCV infection, 28 post-SVR patients, and 12 healthy controls.

**Results:**

In the cross-sectional analysis, HCV eradication was associated with partial restoration of the dysbiotic gut microbiota, including reduced streptococcal overgrowth and an increase in the potentially beneficial genus *Blautia*, approaching levels seen in healthy individuals. Additionally, the aberrant fecal bile acid profile showed rebalancing, accompanied by restored expression of genes involved in the classical pathway of cholic and chenodeoxycholic acid biosynthesis. In the longitudinal study, improvements in liver fibrosis and function – evidenced by decreased Fibrosis-4 index and alanine aminotransferase levels – were significantly correlated with increased abundance of Blautia (*p* <0.0001 and *p* = 0.0344, respectively), suggesting a beneficial role in liver recovery.

**Conclusion:**

The gut-microbiota-liver axis is partially restored following HCV eradication, with recovery from liver damage associated with the resurgence of commensal Lachnospiraceae species.

**Impact and implications:**

This study offers significant insights into the gut-microbiota-liver axis in patients with chronic hepatitis C following a sustained virological response. The findings demonstrate that HCV eradication promotes partial restoration of the dysbiotic gut microbiota, particularly an increase in the beneficial genus *Blautia*, as well as a rebalancing of the fecal bile acid profile. These changes are closely associated with significant improvements in liver fibrosis and function, highlighting a potential role of the gut microbiota in liver recovery and regeneration.

## Highlights

• This study investigates the gut-microbiota-liver axis in patients with chronic hepatitis C following SVR.
• During HCV infection, the classical bile acid biosynthetic pathway is suppressed.
• Intestinal deoxycholic acid levels are restored to normal following the achievement of SVR.
• An improvement in liver fibrosis and function is associated with an increase in *Blautia* species.

## Graphical abstract

**Figure:**
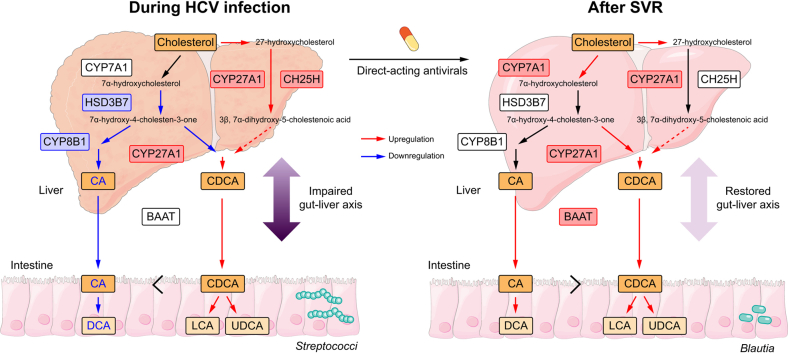

## Introduction

Hepatitis C virus (HCV) infects approximately 58 million people worldwide, causing progressive liver damage that can lead to cirrhosis (LC) and hepatocellular carcinoma (HCC).^1^^,^^2^ The advent of direct-acting antivirals (DAAs) has transformed HCV treatment, achieving sustained virological responses (SVR) in over 90% of patients.^3^ This virological cure reduces the risks of hepatic and extrahepatic complications while improving health-related quality of life.^3^ However, the post-SVR pathophysiological changes, particularly involving the gut-microbiota-liver axis, remain poorly understood.

We have reported that chronic hepatitis C (CHC) is associated with gut dysbiosis, marked by reduced bacterial diversity and compositional shifts, including decreased *Clostridiales* and increased *Streptococci* and *Lactobacilli*.^4^ Additionally, we found HCV infection disrupts bile acid (BA) metabolism through an imbalance between the classical and alternative *de novo* synthesis pathways in the liver. This imbalance not only impairs hepatic function but also alters the gut microbial community, affecting the conversion of primary to secondary BAs. These changes are reflected in reduced fecal deoxycholic acid (DCA) to lithocholic acid (LCA) and ursodeoxycholic acid (UDCA) ratios, as well as decreased hepatic transcription of cytochrome P450 8B1 (CYP8B1), a critical enzyme in cholic acid synthesis. Similar disruptions have been observed in HCV-infected animal models, reinforcing the systemic effects of HCV on the gut-liver axis.^5^

BAs serve as both metabolic regulators and microbial modulators, making them critical components of the gut-microbiota-liver axis.^6^ Through receptors such as FXR (farnesoid X receptor) and TGR5 (Takeda G-protein-coupled receptor 5), BAs regulate inflammation, glucose homeostasis, and lipid metabolism, while acting as antimicrobial agents to shape gut microbiota.^7^ Dysregulated BA metabolism has been implicated in liver disease progression, emphasizing its importance in understanding CHC pathophysiology and recovery post-SVR.^5^^,^^7^^,^^8^

Regarding the gut microbiome in CHC, while some studies have reported improvements in gut microbiota composition post-SVR, including increased short-chain fatty acid-producing bacteria such as *Blautia* and *Bifidobacterium*,^9^^,^^10^ others observed limited or no changes, particularly in patients with advanced liver disease.^11^^,^^12^ These discrepant findings suggest a complicated underlying interaction between the gut microbiota and the liver after SVR.

To address this subject, we conducted a longitudinal observational study, following the cross-sectional investigation, to investigate the recovery of the gut microbiota in conjunction with liver function. Notably, we focused on BA metabolism in the gut microbial community and bile acid biosynthesis in the liver, to gain new insights into the dynamic interaction within the gut-microbiota-liver axis.

## Patients and methods

The study design and methods are detailed in the supplementary materials. This retrospective study included 272 participants: 174 patients with active HCV infection, 75 post-SVR patients, and 23 healthy individuals. Among the patients with HCV, 166 had been previously reported,^4^ and eight new cases were added in this study.

Patients with persistently normal alanine aminotransferase (ALT) values (PNALT) and chronic hepatitis (CH) were classified as CH-HCV, while those with LC and HCC were grouped as LC/HCC-HCV. Similarly, post-SVR patients were categorized into CH-SVR (PNALT and CH) and LC/HCC-SVR (LC and HCC). We analyzed gut microbiome and BA composition across these four groups and healthy individuals.

**Fig. 1:**
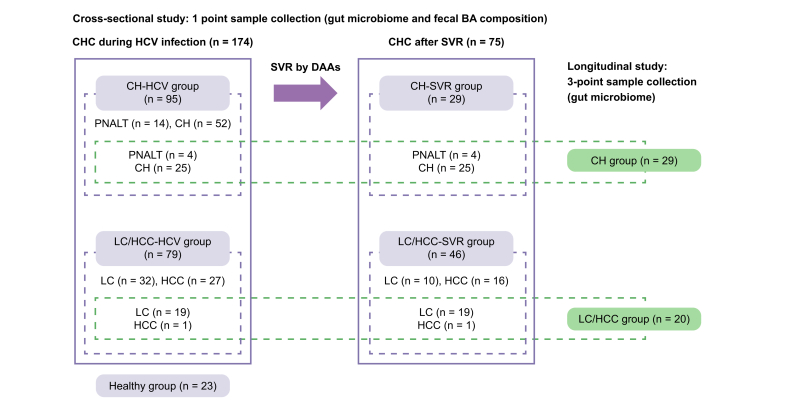
**Study design of the cross-sectional and longitudinal studies**. In the cross-sectional study, a one-point sample was collected from each individual and examined for gut microbiome, fecal BA composition, and gene transcription. Five groups (CH-HCV, LC/HCC-HCV, CH-SVR, LC/HCC-SVR, and healthy groups) were examined. In the longitudinal study, 3-point samples (during HCV infection, SVR24, and SVR48) were collected from each individual and the gut microbiome was examined. Three groups (CH, LC/HCC, and healthy groups) were examined. All cases analyzed in the longitudinal study were also enrolled in the cross-sectional study. BA, bile acid; CH, chronic hepatitis; CH-HCV group, PNALT or CH under HCV infection group; CH-SVR group, PNALT or CH after SVR group; CHC, chronic hepatitis C; HCC, hepatocellular carcinoma in cirrhosis; healthy group, healthy individuals group; LC, cirrhosis; LC/HCC-HCV group, LC or HCC under HCV infection group; LC/HCC-SVR group, LC or HCC after SVR group; PNALT, persistently normal alanine aminotransferase; SVR, sustained virological response.

Additionally, 49 CHC patients from the cross-sectional study were included in a longitudinal analysis to assess gut microbiome changes at 24 and 48 weeks post-SVR (SVR24 and SVR48, respectively). Details are provided in Fig. 1 and Table S1; patient characteristics are summarized in Table 1 (cross-sectional) and Table S2–S3 (longitudinal). Definitions of clinical stages and inclusion/exclusion criteria were described in our previous papers.^4^^,^^5^ All patients diagnosed with HCC had a background of LC.

**Table 1:** Demographics and clinical characteristics of patients during HCV infection (n = 174), after SVR (n = 75), and of healthy individuals (n = 23) in the cross-sectional study.

Characteristics	Category (number of candidates)				
	During HCV infection		After SVR		Healthy group (n = 23)
	CH-HCV group (n = 95)	LC/HCC-HCV group (n = 79)	CH-SVR group (n = 29)	LC/HCC-SVR group (n = 46)	
Gender (M/F)	40/55	39/40	13/16	21/25	15/8
Age (years)	66.3 ± 12.9^a^	71.5 ± 9.3^a,b^	65.2 ± 12.0	70.7 ± 9.1^c^	61.3 ± 8.1^b,c^
PLT (×10^4^/mm^3^)	18.3 ± 5.1^d,e,f^	10.2 ± 44^d,g^	20.3 ± 5.1^e,g,h^	11.6 ± 4.1^f,h^	n.d.
PT (%)	91.2 ± 12.3^i^	78.3 ± 14.9^i,j,k^	99.4 ± 7.4^j^	90.0 ± 13.1^k^	n.d.
ALB (g/dl)	4.1 ± 0.4^l,m^	3.6 ± 0.6^l,n,o^	4.4 ± 0.3^m,n^	4.2 ± 0.5^o^	n.d.
AST (IU/L)	44.4 ± 41.5^p^	53.9 ± 43.2^q,r^	23.4 ± 8.8^p,q^	31.6 ± 19.8^r^	n.d.
ALT (IU/L)	49.7 ± 107.4	38.4 ± 38.4	16.8 ± 9.5	21.4 ± 13.3	n.d.
GGT (IU/L)	33.5 ± 31.9	45.5 ± 42.1	19.0 ± 7.5^s^	58.7 ± 127.5^s^	n.d.
T-Bil (mg/dl)	0.9 ± 0.7	1.3 ± 1.1	0.9 ± 0.7	1.2 ± 1.3	n.d.
AFP (ng/ml)	6.4 ± 14.5	113.5 ± 702.5	3.2 ± 1.8	9.3 ± 20.5	n.d.
PIVKA-II (mAU/ml)	20.7 ± 14.3	2,145.9 ± 17,360.7	30.8 ± 58.4	109.0 ± 502.9	n.d.
FIB-4 index	3.3 ± 2.2^t,u^	7.4 ± 4.1^t,v,x^	2.0 ± 1.0^v,y^	5.1 ± 3.2^u,x,y^	n.d.

AFP, alpha fetoprotein; ALB, serum albumin; ALT, alanine aminotransferase; AST, aspartate aminotransferase; FIB-4 index, fibrosis-4 index; GGT, γ-glutamyltransferase; healthy, healthy individuals; n.d., not determined; PIVKA-II, protein induced by vitamin K absence or antagonist-II; PLT, platelet count; PT, prothrombin time; T-Bil, total bilirubin.

Continuous data are expressed as means ± SD. Superscript letters indicate a significant difference in one-way ANOVA followed by Tukey–Kramer post analysis (*p* <0.001: e, f, g, h, j, k, l, m, p, u, v, w and x; *p* <0.01: d; *p* <0.05: a, b, c, I, n, o, q, r, s and t). There is no significant difference in sex ratio between each CHC group and the healthy group.

The procedures for gut microbiome analysis, including DNA extraction from stool samples, 16S rRNA gene amplification, high-throughput sequencing, and data processing using QIIME2 (qiime2-2023.2, https://qiime2.org),^13^ are detailed in the supplementary material. Summary statistics for 16S rRNA profiling, amplicon sequence variants (ASVs), and taxonomic classifications are provided in Table S4–S8. α- and β-diversity indices of gut microbiota across clinical stages (pre- and post-SVR) and comparisons with healthy individuals are described in the supplementary material. Additionally, longitudinal microbiome changes post-SVR and their association with liver function were analyzed at the genus level in CH and LC/HCC groups, as detailed in the supplementary material.

Among the 272 individuals analyzed for gut microbiota, BA analysis was performed on 176 patients – including 100 with active HCV infection, 53 post-SVR, and 23 healthy individuals – with sufficient sample volumes. Fifteen major fecal BAs were quantified using high-performance liquid chromatography coupled with triple quadrupole mass spectrometry (LCMS-8050, SHIMADZU CORPORATION), as previously described.^5^ Details of fecal BA profile comparisons across clinical stages and healthy individuals are provided in the supplementary material.

Whole-transcriptome sequencing (RNA-seq) procedures have been previously described.^5^ We enrolled 65 patients with HCV infection, 28 post-SVR patients, and 12 age-matched healthy liver controls from organ donors. RNA-seq data were obtained from the Sequence Read Archive, and clinical and pathological data are summarized in Table S9.

Details on statistics and accession numbers for the 16S rRNA gene sequence data are provided in the supplementary material. All participants provided written informed consent, and the study was approved by institutional ethics committees in accordance with the Declaration of Helsinki.

## Results

### Clinical characteristics of patients with active HCV and healthy individuals

In the cross-sectional study, 32 of the LC/HCC-HCV group (40.5%) had decompensated cirrhosis. Advanced investigations beyond ultrasonography included biopsy (n = 4), MRI (n = 19), CT (n = 12), elastography plus MRI (n = 7), MRI plus CT (n = 16), and MRI plus biopsy (n = 21). Demographic and clinical differences among groups are summarized in Table 1, with no significant age or sex differences between CH-HCV, CH-SVR, and healthy groups.

In the longitudinal study, 49 patients with CHC (29 CH group and 20 LC/HCC group) were enrolled. Sample size calculations confirmed sufficient statistical power to monitor *Blautia* abundance changes. The minimum required sample sizes – 28 for CH and 15 for LC/HCC – were determined based on mean and variance in pre- and post-SVR groups (details in the supplementary materials). Significant demographic and clinical differences are shown in Table S3.

### Cross-sectional findings

#### Recovery of gut microbial diversity (α-diversity) within individuals after SVR

**Fig. 2:**
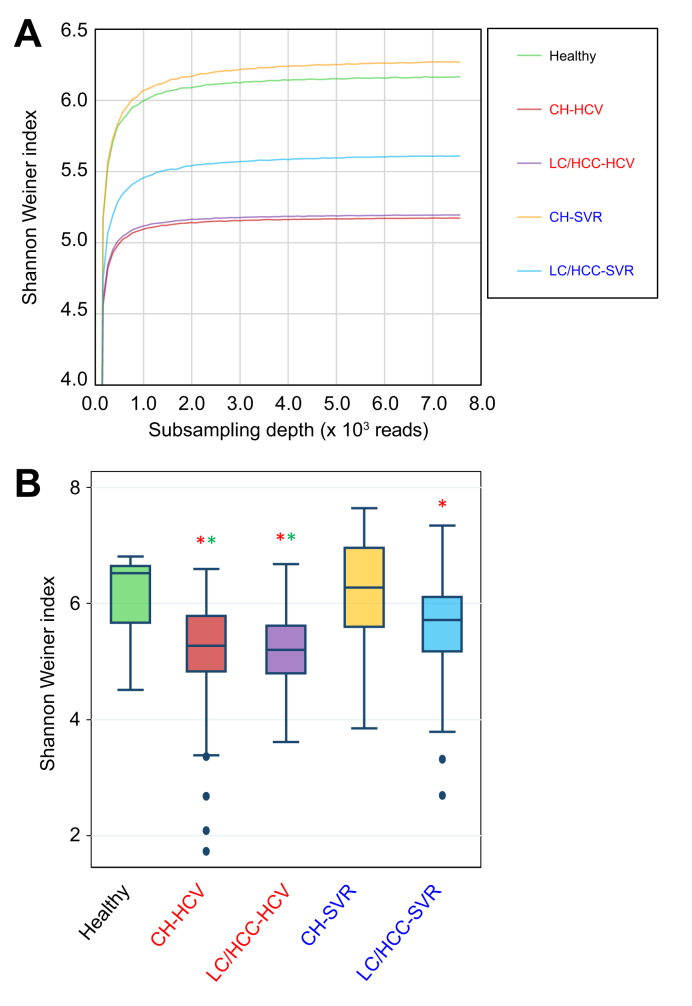
**α-diversity of the gut microbiome of patients during HCV infection and after SVR**. (A) Rarefaction curve of the Shannon–Wiener index with increasing sampling depth. (B) Box plot showing Shannon–Wiener indices for each group at a sampling depth of 7,500 reads, including the minimum, maximum, quartiles, median, and outliers. Statistical differences from the healthy control were assessed using non-parametric Dunnett's test. Red and green asterisks indicate significant differences (*p* <0.05) with sufficient power (1-β >0.8). CH, chronic hepatitis; CH-HCV, PNALT or CH under HCV infection group; CH-SVR, PNALT or CH after SVR group; CHC, chronic hepatitis C; HCC, hepatocellular carcinoma in cirrhosis; healthy, healthy individuals group; LC, cirrhosis; LC/HCC-HCV, LC or HCC under HCV infection group; LC/HCC-SVR, LC or HCC after SVR group; PNALT, persistently normal alanine aminotransferase; SVR, sustained virological response.

The Shannon–Wiener index, which is a measure of taxonomic richness and evenness within an individual, was lower in the CH-HCV and LC/HCC-HCV groups than the healthy group, suggesting an imbalanced microbiome damaged during HCV infection, so-called dysbiosis. After SVR, α-diversity recovered to a level similar to the healthy group, which was more obvious in the CH-SVR than the LC/HCC-SVR group, suggesting the liver damage is even worse in the LC/HCC groups (Fig. 2A,B).

#### Recovery of the gut microbial community after SVR

**Fig. 3:**
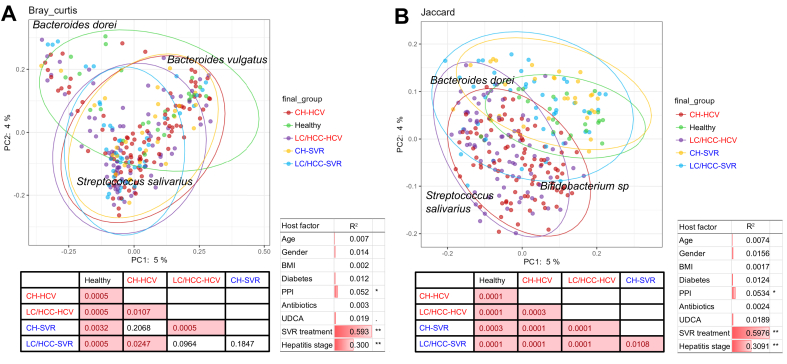
**β-diversity of the gut microbiome of patients during HCV infection and after SVR**. PCoA plots showing the microbiome beta-diversity of samples from patients with CHC at different clinical stages and healthy individuals. Diversities were calculated using (A) Bray-Curtis and (B) Jaccard distances based on ASV composition dissimilarities. Ellipses indicate 95% confidence intervals for group distributions, with statistical differences shown below the plots (*p* values from Permanova analysis). Correlations of host factors to ordination are listed beside the plots as r^2^ values, with single asterisks (*p* <0.05) and double asterisks (*p* <0.001) indicating significance. ASV, amplicon sequence variant; CH, chronic hepatitis; CH-HCV, PNALT or CH under HCV infection group; CH-SVR, PNALT or CH after SVR group; CHC, chronic hepatitis C; HCC, hepatocellular carcinoma in cirrhosis; healthy, healthy individuals group; LC, cirrhosis; LC/HCC-HCV, LC or HCC under HCV infection group; LC/HCC-SVR, LC or HCC after SVR group; NMDS, non-metric multidimensional scaling; PCoA, principal component analysis; PNALT, persistently normal alanine aminotransferase; SVR, sustained virological response.

Gut microbial community structure was compared among patients with CHC at different clinical stages and healthy individuals using Bray-Curtis and Jaccard distances, calculated from ASV composition data. Principal coordinate analysis based on these matrices showed distinct clustering of patients with CHC and healthy individuals (Fig. 3A,B). Jaccard distance indicated post-SVR samples clustered with healthy individuals, suggesting gut microbiome recovery after HCV eradication (Fig. 3B).

*Streptococcus salivarius*, known to overgrow in association with HCV infection, was identified as a characteristic microbiome component in patients with CHC (Figs. 3A,B). Other physiological factors – diabetes, obesity, and antibiotic use – showed weaker correlations with microbiome composition (Fig. 3A,B). No participants reported alcohol consumption or selective eating habits. Proton pump inhibitor administration, as reported,^14^^,^^15^ influenced gut microbiome structure but had a minor impact compared to HCV eradication. These findings indicate that microbiome alterations were primarily driven by HCV infection and eradication, with minimal confounding effects (Fig. 3A,B; Table S10 and S11).

#### Changes in gut microbiome during HCV infection and after its eradication

**Fig. 4:**
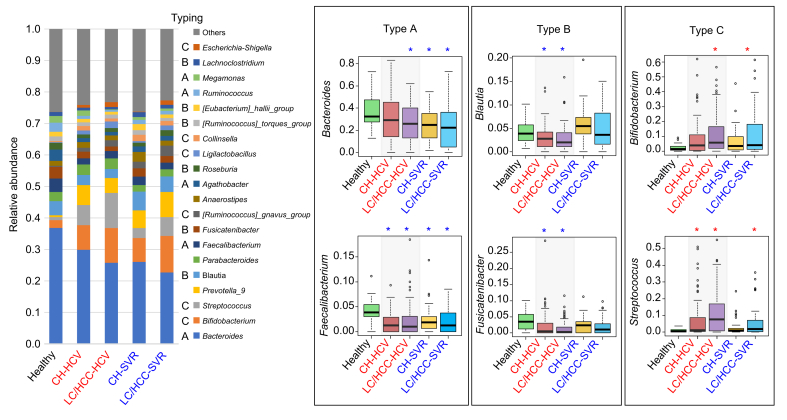
**Impact of HCV infection and eradication on gut microbiota**. The genus composition of each sample was averaged within each group and displayed in the stacked bar chart. The relative abundance of each genus at each hepatitis stage was compared to the healthy group and categorized as follows: Type A decreased during HCV infection and did not recover after eradication. Type B decreased during HCV infection but tended to recover after eradication. Type C increased during HCV infection and showed a decreasing trend after eradication. Representative genera for each type are shown in the box plot, which includes minimum, maximum, quartiles, median, and outliers. Asterisks (∗) indicate significant differences (*p* <0.05) from the healthy group (red: increase, blue: decrease) as determined by non-parametric Dunnett’s test. CH, chronic hepatitis; CH-HCV, PNALT or CH under HCV infection group; CH-SVR, PNALT or CH after SVR group; CHC, chronic hepatitis C; HCC, hepatocellular carcinoma in cirrhosis; healthy, healthy individuals group; LC, cirrhosis; LC/HCC-HCV, LC or HCC under HCV infection group; LC/HCC-SVR, LC or HCC after SVR group; SVR, sustained virological response.

Genus compositions of fecal samples from patients with CHC at various clinical stages were compared to those of healthy individuals (Fig. 4). There were three genus groups, classified according to their changing patterns, as follows: Type A comprises genera which decreased during HCV infection but did not recover after HCV elimination. The most dominant genus, *Bacteroides*, and *Faecalibacterium*, known as a beneficial butyrate producer, belong to this type. Type B comprises genera which decreased during HCV infection but recovered after HCV eradication. *Blautia*, *Fusicatenibacter*, and *Roseburia*, fall into this category. The post-SVR recovery of these genera was more complete in the CH group than the LC/HCC group. Type C comprises genera which increased with HCV infection. *Streptococcus* and *Bifidobacterium* belong to this type. The increase in these two genera was more evident in the LC/HCC group than the CH group. *Streptococcus* tended to decrease after HCV eradication, while the level of *Bifidobacterium* did not change after HCV eradication (Fig. 4).

#### Rebalance of fecal BA composition after HCV eradication

**Fig. 5:**
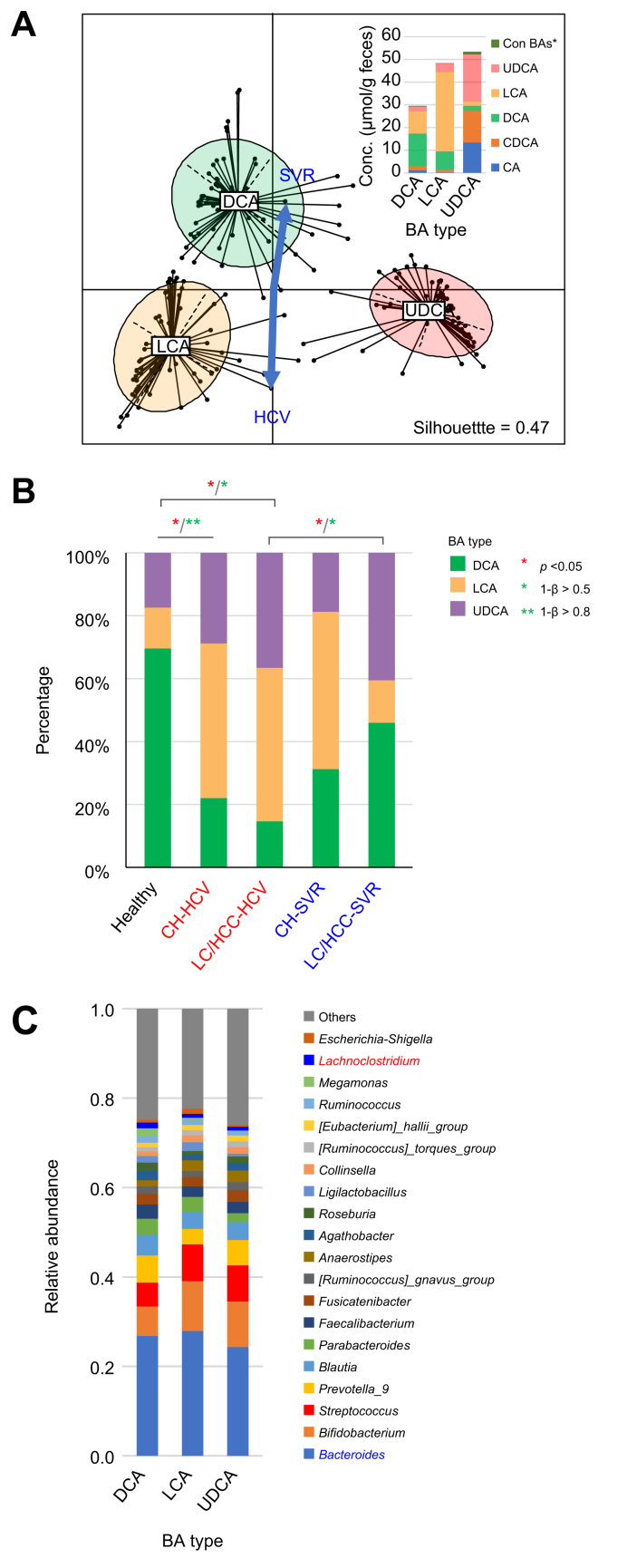
**Fecal BA typing of patients with CHC at different clinical stages and healthy individuals**. (A) Ordination and clustering of samples based on fecal BA composition, defining three clusters: DCA (n = 57), LCA (n = 65), and UDCA (n = 54). Blue arrows represent host factors significantly correlated with the ordination. Euclidean distances were calculated from the relative abundances of 15 major BAs, and PAM clustering identified three clusters based on the highest Calinski-Harabasz index and a silhouette width of 0.47. An inset bar chart shows the BA composition for each type. (B) Distribution of healthy individuals and patients with CHC across the three BA types. Red asterisks denote significant group differences (*p* <0.05, McNemar’s test). Single and double green asterisks indicate statistical power levels of 1-β <0.5 and 1-β <0.8, respectively. (C) Average genus composition within each BA type. Genera in red and blue indicate significantly higher and lower abundances in the DCA type compared to others (pairwise Wilcoxon rank-sum test with BH adjustment, *p* <0.05). ASV, amplicon sequence variants; BA, bile acid; CA, cholic acid; CDCA, chenodeoxycholic acid; CH, chronic hepatitis; CH-HCV, PNALT or CH under HCV infection group; CH-SVR, PNALT or CH after SVR group; CHC, chronic hepatitis C; DCA, deoxycholic acid; HCC, hepatocellular carcinoma in cirrhosis; healthy, healthy individuals group; LCA, lithocholic acid; LC, cirrhosis; LC/HCC-HCV, LC or HCC under HCV infection group; LC/HCC-SVR, LC or HCC after SVR group; SVR, sustained virological response; UDCA, ursodeoxycholic acid.

Fecal BA composition, including 15 BA molecules, was analyzed in 176 samples from patients with CHC (at different clinical stages) and healthy individuals. As in the previous study, DCA ratios decreased in CH and LC/HCC groups but partially recovered post-SVR (Fig. S1). While LCA and UDCA abundance showed no significant difference from healthy controls, their variance increased (Fig. S1). Clustering analysis grouped BA profiles into three types dominated by DCA, LCA, or UDCA (Fig. 5A, inset). Although total BA levels were higher in LCA and UDCA types, the difference was not statistically significant. Healthy individuals predominantly had the DCA type, whereas CH and LC/HCC groups showed higher proportions of LCA or UDCA (Fig. 5B), contributing to variance in LCA and UDCA levels (Fig. S1B). Post-SVR, BA profiles tended to shift toward DCA, especially in the LC/HCC group (Fig. 5A). Other physiological factors had no statistical impact on BA typing (Fig. S2A).

Few bacterial genera differed significantly among BA types; *Bacteroides* was lower, and *Lachnoclostridium* was higher in the DCA type (Fig. 5C). LCA and UDCA types showed fewer ASVs, particularly within *Lachnospiraceae* (Fig. S3). Correlation analysis revealed genera within *Clostridia*, including *Agathobacter*, *Megamonas*, *Faecalibacterium*, and *Lachnoclostridium*, positively associated with DCA, whereas *Streptococcus* showed a negative correlation (Fig. S2A). *Lachnoclostridium*, including *L. scindens* (a secondary BA producer), had a strong positive correlation with DCA. No genera showed significant correlation with LCA, although Blautia, which is classified as B-type, exhibited the strongest negative coefficient (Fig. 4).

#### Comparison of the expression of BA metabolism-related genes, before and after HCV eradication

This study analyzed gene expression levels of key enzymes involved in BA biosynthesis using RNA-seq data, comparing individuals with healthy livers, patients with HCV infection, and those post-SVR.

**Fig. 6:**
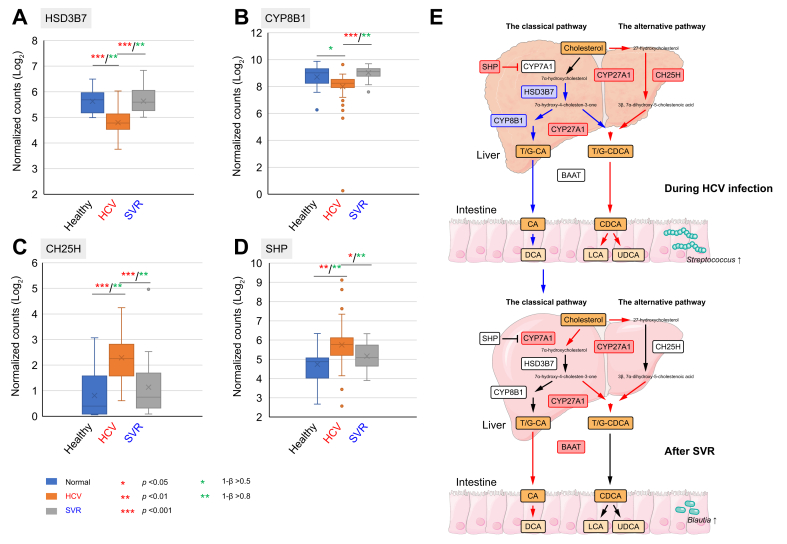
**Changes in the gene expression levels of enzymes involved in BA biosynthesis in the liver after SVR**. Gene expression in the liver related to BA metabolism analyzed by RNA-seq in 65 patients with CHC (F0-2 [n = 22], F3-4 [n = 43]) and 12 healthy individuals. Red and green asterisks indicate significant differences and statistical power, respectively. (A) *HSD3B7*, (B) *CYP8B1*, (C) *CH25H,* and (D) *SHP.* (E) Comparison of BA dysmetabolism during HCV infection and after SVR. Red characters and arrows represent upregulated genes; blue denote downregulated genes. Statistical analysis was performed using the Kruskal-Wallis test, followed by Steel-Dwass test and Bonferroni adjustment. BA, bile acid; BAAT, bile acid-CoA amino acid N-acyltransferase; CA, cholic acid; CDCA, chenodeoxycholic acid; CH25H, cholesterol 25-hydroxylase; CHC, chronic hepatitis C; CYP27A1, sterol 27-hydroxylase; CYP7A1, cholesterol 7α-hydroxylase; CYP7B1, oxysterol 7α-hydroxylase; CYP8B1, cytochrome P450 8B1; DCA, deoxycholic acid; Healthy, individuals with healthy liver; HSD3B7, 3 beta-hydroxysteroid dehydrogenase type 7; LCA, lithocholic acid; RNA-Seq, transcriptional analysis; SHP, small heterodimer partner; SVR, sustained virological response; UDCA, ursodeoxycholic acid.

In the classical pathway, cholesterol 7-alpha-hydroxylase (CYP7A1), which converts cholesterol to 7α-hydroxycholesterol,^16^ was upregulated in patients with CHC during HCV infection and remained elevated post-SVR, compared to individuals with healthy livers (Fig. S4A). Conversely, 3 beta-hydroxysteroid dehydrogenase type 7 (HSD3B7) and CYP8B1, downstream of CYP7A1, were downregulated during HCV infection but recovered post-SVR to levels similar to those in individuals with healthy livers (Figs. 6A,B). Sterol 27-hydroxylase, downstream of CYP8B1, exhibited greater upregulation post-SVR compared to during HCV infection (Fig. S4B).

In the alternative pathway, cholesterol 25-hydroxylase was upregulated in patients during HCV infection but returned to levels similar to those of individuals with healthy livers after SVR (Fig. 6C). Small heterodimer partner, which mediates FXR signaling to CYP7A1,^17^ was significantly upregulated in patients during HCV infection, and recovered after SVR to levels similar to the those in individuals with healthy livers (Fig. 6D). Fig. 6 illustrates that BA biosynthetic pathways toward DCA recover, whereas the pathway leading to LCA via chenodeoxycholic acid remains upregulated post-SVR.

All comparisons with *p* <0.05 exhibited 1 − β >0.8, confirming robust statistical power for this RNA-seq analysis. Fig. 6E presents a simplified BA metabolic pathway in patients during HCV infection and post-SVR.

### Longitudinal changes of the gut microbiome before and after HCV eradication and their association with liver fibrosis and inflammatory indicators

**Fig. 7:**
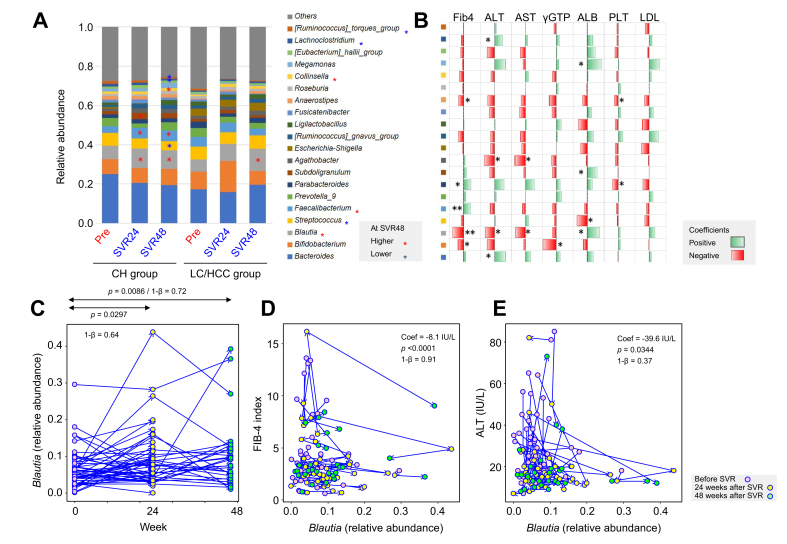
**Longitudinal changes of fecal microbiota before and after HCV eradication and their association with liver function indicators**. (A) Genus-level gut microbiota changes after HCV elimination in CH and LC/HCC groups. Red and blue asterisks indicate significantly higher and lower levels compared to pre-eradication (Wilcoxon signed-rank test, *p* <0.05). (B) Correlation of genus abundance changes with liver function indicators after HCV eradication. Green and red bars denote positive and negative coefficients, respectively. Statistically significant correlations are marked by asterisks (∗*p* <0.05, ∗∗*p* <0.001, GEE analysis). Genera correspond to Fig. 7A. (C) Relative abundance of *Blautia* at SVR24 and SVR48. (D) Changes in FIB-4 index and *Blautia* abundance after SVR. (E) Changes in ALT levels and *Blautia* abundance after SVR. Pink, yellow, and green dots represent data before SVR, and at SVR24 and SVR48, respectively. Statistical power (1-β) is shown in the figure. ALB, serum albumin; ALT, alanine aminotransferase; AST, aspartate aminotransferase; CH, chronic hepatitis; CHC, chronic hepatitis C; FIB-4, fibrosis-4 index; GGT, gamma-glutamyltransferase; HCC, hepatocellular carcinoma; LDL, low density lipoprotein cholesterol; LC, cirrhosis; PNALT, persistently normal alanine aminotransferase; PLT, platelet count; Pre, the status of HCV infection; SVR, sustained virological response; SVR24, SVR at 24 weeks after treatment end; SVR48, SVR at 48 weeks after treatment end.

In the CH group, *Blautia* and *Faecalibacterium* increased significantly at SVR24 and SVR48 (*p* <0.05), *Subdoligranulum* at SVR24 (*p* <0.05), and *Collinsella* at SVR48 (*p* <0.05). Meanwhile, *Streptococcus*, *[Eubacterium]_hallii_group*, and *[Ruminococcus]_torques_group* decreased significantly at SVR48 (each *p* <0.05). In the LC/HCC group, only *Blautia* increased significantly at SVR48 (*p* <0.05), with no significant changes in other species (Fig. 7A).

Fig. 7B illustrates correlations between bacterial abundance changes and liver function parameters. *Blautia* negatively correlated with the Fibrosis-4 index (FIB-4) (*p* <0.001, 1-ß = 0.91), ALT (*p* <0.05, 1-ß = 0.37), aspartate aminotransferase (AST) (*p* <0.05, 1-ß = 0.41), and positively with serum albumin (ALB) (*p* <0.05, 1-ß = 0.46), indicating its association with liver function recovery. *Agathobacter* showed negative correlations with ALT and AST (both *p* <0.05, 1-ß = 1.00), but not FIB-4, while *Streptococcus* negatively correlated only with ALB (*p* <0.05, 1-ß = 0.87). *Faecalibacterium* had a positive correlation with FIB-4 (*p* <0.05, 1-ß = 0.82), despite its post-SVR increase.

Fig. S6 presents ASV-level data, showing ASVs in *Lachnospiraceae* (including *Blautia* and *Lachnoclostridium*) correlated negatively with FIB-4, ALT, AST, and γ-glutamyltransferase. Individual-level changes in *Blautia* and its correlation with liver fibrosis and inflammation are depicted in Fig. 7C–E and Fig. S4. Post-SVR, *Blautia* increased most notably at 24 weeks in patients with initially lower levels (Fig. 7C–E and Fig. S5). Its increase strongly correlated with reductions in FIB-4 (coefficient = -0.081 per 1% increase), ALT (coefficient = -0.396 IU/L per 1% increase), AST (coefficient = -0.434 IU/L per 1% increase, Fig. S5A), and an ALB rise (coefficient = 0.0147 g/dl per 1% increase, Fig. S5B), indicating that improvements in liver fibrosis and inflammation are linked to the increase in *Blautia*.

## Discussion

The cross-sectional observational study indicated that HCV eradication tended to lead to recovery of the gut microbiota from the dysbiotic state observed in patients with CHC. Although the microbiome structures of patients after SVR did not fully overlap with those of the healthy group (Fig. 3), the reduced α-diversity, indicative of a dysbiotic state, had recovered to healthy levels in the CH group and partially recovered in the LC/HCC group (Fig. 2). The recovery pattern of gut bacteria depended on the genera (Fig. 4). *Blautia* and *Fusicatenibacter* recovered to levels statistically comparable to those of healthy controls in both the CH and LC/HCC groups, whereas the increase in *Bifidobacterium* and *Streptococcus* and the decrease in *Bacteroides* and *Faecalibacterium* did not normalize. These findings more or less align with several previous reports.[10], [11], [12]^,^^18^

The subsequent longitudinal analysis revealed an increase in potentially beneficial bacteria, such as *Faecalibacterium* and *Blautia*, after HCV eradication. While numerous studies have highlighted the positive effects of *Faecalibacterium* on host health,^19^ this study identified *Blautia*, rather than *Faecalibacteria*, as a key commensal genus associated with improved liver function and reduced fibrosis. *Blautia* is notably abundant in the Japanese gut microbiome and plays pivotal roles in the health of the host.^20^ For example, a Japanese cohort study demonstrated an inverse correlation between *Blautia* abundance and obesity and type 2 diabetes mellitus, while an animal study suggested that *Blautia* and its metabolites ameliorate these metabolic disorders through their anti-inflammatory properties and modulation of the gut environment and lipid metabolism.^21^^,^^22^ The anti-inflammatory activity of *Blautia* may support the recovery of liver function and tissue repair, while the lower level of *Blautia* could compromise the liver defense mechanism in CHC. In addition to the microbiome analysis, we performed an intensive fecal metabolome analysis of BA composition, evaluated in conjunction with the liver tissue RNA-seq data analysis, to capture the status of *de novo* BA biosynthesis in the livers of patients with CHC. Our BA typing analysis revealed the shift from DCA type to LCA type in patients with HCV infection and the recovery to the DCA type after HCV eradication (Fig. 5, Fig. 6).

RNA-seq analysis of the liver biopsy samples further demonstrated recovery of the balance between the classical pathway and the alternative pathway.^17^ As shown in Fig. S2, a great number of Clostridia species, which are mostly commensal bacteria generally observed in healthy microbiota and found to be reduced in patients with HCV in our previous study,^4^ correlated with the DCA type, while non-Clostridia group, including *Bacteroides*, *Bifidobacterium*, *Streptococcus*, and *Escherichia-Shigella*, inversely correlated with the DCA type. Because 7α-dehydroxylation probably occurs evenly on cholic acid and chenodeoxycholic acid through the activity of certain bacterial enzymes, it is thought that the shift of BA-type is caused by the alteration of liver function, as shown by the RNA-seq data. However, the finding that the total concentration of BAs in DCA-type feces is lower than in LCA/UDCA-type feces (inset in Fig. 5A) and that the number of ASVs observed in the DCA type is higher than in the LCA/UDCA type (Fig. S3) suggest the presence of antimicrobial pressure, attributable to the high concentration of BAs in patients with CHC. Taken together, the changes in gut microbiota associated with shifts in BA type appear to result from the combined effects of both quantitative and qualitative changes in BAs.

In the case of MASLD (metabolic dysfunction-associated steatotic liver disease), a reduction of 7α-dehydroxylation leading to an increased ratio of primary to secondary BAs has been reported, in contrast to the imbalance between DCA and LCA found in this CHC study.[23], [24], [25] This is associated with gut microbiome dysbiosis, where the gut commensal population, including Clostridiales, decreases. Such dysbiosis is often observed in non-communicable diseases, suggesting a link between exposome factors, particularly unhealthy diet, and the exacerbation of the gut environment, such as an increase in bactericidal BA levels.^24^ On the other hand, a study of patients infected with HBV indicated that a higher level of DCA was inversely associated with HCC risk,^25^ in agreement with this study. The impact of the reduction of DCA caused by viral infection of liver tissues represents a different aspect of hepatitis progression, compared to hepatitis in non-communicable diseases.

Collectively, we propose the following model for the recovery of the liver-gut microbiome axis following SVR. HCV-damaged liver function leads to an imbalance in *de novo* BA biosynthesis, which recruits an LCA-type gut microbiome. This imbalance increases the level of highly hydrophobic and cytotoxic LCA and decreases the abundance of the commensal Clostridiales group, which has health-promoting effects (such as short-chain fatty acid production), exacerbating liver damage.^26^ Following HCV eradication, the liver-gut microbiome axis begins to restore itself. *De novo* BA biosynthesis is rebalanced in the liver and the gut microbiota recovers from dysbiosis, thereafter improving liver function. The observed increase in *Blautia* levels during recovery is associated with reduced hepatic damage, suggesting a potential protective role in this context.

Our study has several limitations. First, the patients with CHC providing fecal samples and those providing liver tissues were not entirely the same, and RNA-seq data from patients with CHC and individuals with healthy livers were partially sourced from a database, limiting the feasibility of comprehensive correlation analyses. Second, in the longitudinal study, the sample size at SVR48 was limited because many patients achieving SVR24 returned to their referring institutions. Third, we acknowledge that the use of separate cohorts for microbiome, BA, and hepatic transcriptomic analyses represents a significant limitation of this study, as it precludes direct cross-omic correlation and restricts the depth of mechanistic inference. This constraint limits our ability to derive integrative insights into gut-liver axis dynamics. Nevertheless, our findings provide valuable foundational knowledge, laying the groundwork for more comprehensive, multi-layered analyses in future research.

To refine future integrative analyses, we propose computational strategies to mitigate cohort separation effects. Bayesian hierarchical modeling and batch effect correction may help harmonize data from distinct biological sources, while emerging artificial intelligence-driven integration techniques offer promising approaches for aligning heterogeneous omic layers. Specifically, computational frameworks such as Multi-Omics Factor Analysis Plus (MOFA+) and Data Integration Analysis for Biomarker discovery using Latent cOmponents (DIABLO) are designed to infer cross-modal associations, even in partially matched or non-overlapping datasets. Implementing such approaches in future studies could enhance our ability to uncover mechanistic links between microbiome dynamics, BA metabolism, and hepatic gene expression. Despite these limitations, the proposed computational strategies offer a promising path toward bridging multi-omic gaps. To that end, we are actively planning prospective studies that incorporate harmonized sampling designs to enable fully integrative analyses.

In conclusion, HCV eradication corrects the imbalance of BA biosynthesis and allows the gut microbiome composition to recover, becoming closer to that of healthy individuals, with variations depending on the prior clinical stage of CHC. A key finding is the recovery of *Blautia* levels after HCV eradication, which is associated with improvements in liver fibrosis and inflammation markers. These results highlight *Blautia’*s potential as a live biotherapeutic product to aid the recovery of liver function alongside DAA therapy. Additionally, *Blautia* may serve as a biomarker for predicting liver fibrosis and inflammation outcomes, warranting further investigation in large-scale observational studies of DAA-treated patients. This study underscores the importance of targeting the gut microbiome to support liver recovery in patients with CHC.

## Abbreviations

ALB, serum albumin; ALT, alanine aminotransferase; ASVs, amplicon sequence variants; AST, aspartate aminotransferase; BA, bile acid; CH, chronic hepatitis; CH-HCV, PNALT or CH under HCV infection group; CH-SVR, PNALT or CH after SVR group; CHC, chronic hepatitis C; CYP7A1, cholesterol 7-alpha-hydroxylase; CYP8B1, cytochrome P450 8B1; DAAs, direct-acting antivirals; DCA, deoxycholic acid; FIB-4, Fibrosis-4 index; HCC, hepatocellular carcinoma; HSD3B7, 3 beta-hydroxysteroid dehydrogenase type 7; LC, cirrhosis; LC/HCC-HCV, LC or HCC under HCV infection group; LC/HCC-SVR, LC or HCC after SVR group; LCA, lithocholic acid; PNALT, persistently normal ALT values; RNA-seq, whole-transcriptome sequencing; SVR, sustained virological response; SVR24, SVR at 24 weeks after treatment end; SVR48, SVR at 48 weeks after treatment end; UDCA, ursodeoxycholic acid.

## Financial support

This research was supported by AMED under Grant Number JP24fk0210103 and JP25fk0210172, the Ministry of Education, Culture, Sports, Science, and Technology (22K08037), and grant-in-aid for research from Nagoya City University.

## Authors’ contributions

Study concept and design (TI, JN, HM, AT, KK, YT), acquisition of samples (TI, JN, KM, HK, HW, GS, YK, TI, SK, SM, KK, TW, EI, RS, YH, TW, ST, HY), analysis and interpretation of data (TI, JN, HM, MT, DN, MO, YF, RM, YS), drafting of the manuscript (TI, YF, MO, JN), critical revision of the manuscript for important intellectual content (all authors).

## Data availability

The data that support the findings of this study are available from the corresponding author upon reasonable request.

## Conflict of interest

Lecture Fees: AbbVie GK, Gilead Sciences, Inc. (Satoru Kakizaki), AbbVie GK, Gilead Sciences, Inc., Chugai Pharmaceutical Co., Ltd., ASKA Pharmaceutical Holdings Co., Ltd., OTSUKA Pharmaceutical Co., Ltd, Takeda Pharmaceutical Co., Ltd, GlaxoSmithKline PLC., AstraZeneca, Eisai, HU frontier (Yasuhito Tanaka). Consigned/Joint Research Expenses: Fujirebio, Inc., Sysmex Corporation (Takako Inoue and Yasuhito Tanaka), AbbVie GK., GlaxoSmithKline PLC., Gilead Sciences, Inc., Janssen Pharmaceutical K.K. (Yasuhito Tanaka). Scholarship donations: AbbVie GK., OTSUKA Pharmaceutical Co., Ltd (Yasuhito Tanaka).

Please refer to the accompanying ICMJE disclosure forms for further details.
