# Supplementary material for: Restoration of the gut-microbiota-liver axis after hepatitis C virus eradication
Source: JHEP Rep. 2025 Jun 24;7(9):101494. doi: 10.1016/j.jhepr.2025.101494 (PMC12355056; doi:10.1016/j.jhepr.2025.101494)
Supplement: Multimedia component 1 [file mmc1.pdf]

Restoration of the gut-microbiota-liver axis after hepatitis C virus eradication

Takako Inoue, Jiro Nakayama, Hiroshi Mori, Masaru Tanaka, Daisuke Nakagawa, Masaya Ohnishi, Yui Funatsu, Kei Moriya, Hideto Kawaratani, Hisayoshi Watanabe, Goki Suda, Yasuteru Kondo, Tatsuya Ide, Satoru Kakizaki, Satoshi Miuma, Atsushi Suetsugu, Kazuhito Kawata, Takao Watanabe, Etsuko Iio, Rie Momoda, Yutaka Suzuki, Akira Sakamaki, Tsunamasa Watanabe, Takehisa Watanabe, Katsuya Nagaoka, Yoichi Hiasa, Shuji Terai, Hitoshi Yoshiji, Atsushi Toyoda, Ken Kurokawa, Yasuhito Tanaka

Table of contents

Supplementary methods……………………….…………………………………2

Supplementary figures…………………...8

Supplementary tables..16

Supplementary references……………………………………..……..…………24

Supplementary methods

Patients enrolled into the study

The CHC patients (any HCV genotype) were enrolled at Nagoya City University Hospital (Aichi, Japan), Nara Medical University Hospital (Nara, Japan), Gifu University Hospital (Gifu, Japan), Kurume University Hospital (Fukuoka, Japan), Gunma University Hospital (Gunma, Japan), Nagasaki University Hospital (Nagasaki, Japan), Hamamatsu University Hospital (Shizuoka, Japan), Ehime University Hospital (Ehime, Japan), Niigata University Medical and Dental Hospital (Niigata, Japan), St. Marianna University Hospital (Kanagawa, Japan) from October 2013 to March 2022.

Enrolled patients had not taken ursodeoxycholic acid (UDCA) within the 6 months prior to sampling for this study. The inclusion and exclusion criteria, and the definition of each clinical stage in CHC, have been described in our previous papers [1, 2]. Fecal samples were used for gut microbiome analysis and the analysis of BA profiles, and the methods used are described below.

Healthy individuals enrolled for this study

The sampling from healthy individuals was performed as a part of the Asian Microbiome Project that investigates the basal microbiota of Japanese people. The inclusion and exclusion criteria have been described in our previous papers [1, 2].

Study design

This study was conducted retrospectively. Firstly, the cross-sectional study was performed to compare the fecal bacterial compositions of 272 subjects (174 CHC patients with HCV infection, 75 patients after SVR, and 23 healthy individuals). Of them, 166 CHC patients with HCV infection and 23 healthy individuals who had participated in our previous study [1] and their 16S rRNA sequence data were combined into this study. The details are shown in **Fig. 1 and Table S1**. In the cross-

sectional study, the patients who had achieved HCV eradication at least 24 weeks previously were defined as the patients after SVR. Regarding the patients with HCV infection, PNALT (n = 18) plus CH (n = 77) were defined together as the CH-HCV group (n = 95), and patients with LC (n = 51) and HCC (n = 28) were defined together as the LC/HCC-HCV group (n = 79). Regarding the patients after SVR, PNALT (n = 4) and CH (n = 25) were defined together as the CH-SVR group (n = 29), and patients with LC (n = 29) and HCC (n = 17) were defined together as the LC/HCC-SVR group (n = 46). We compared the gut microbiomes and BA composition of these four groups (CH-HCV, LC/HCC-HCV, CH-SVR, LC/HCC-SVR groups) and the healthy individuals.

Subsequently, 49 CHC patients enrolled in the cross-sectional study were also enrolled in the longitudinal study. In the longitudinal study, the gut microbiome during HCV infection was compared to that at 24 and 48 weeks after SVR. Samples obtained 48 weeks after SVR were provided by 29 of the 49 CHC patients. The number of subjects investigated in the longitudinal study satisfied the power of size; the minimum numbers of subjects were estimated to be 28 for the LC/HCC group and 15 for the CH group, by a power analysis using the cross-sectional data (**Fig. 4**) of mean and variance of abundance of genus *Blautia*, before and after SVR in LC/HCC and CH groups (GPower 3.1, a priori test in the Wilcoxon signed-rank matched-pairs test, a statistical power (1-β) of 0.8 and a type I (α) error rate of 0.05) [3]. The details of the subjects are shown in **Table S2**. The patients with PNALT (n = 4) and CH (n = 25) were defined together as the CH group (n = 29), and the patients with LC (n = 19) and HCC (n =1) were defined together as the LC/HCC group (n = 20) and we compared the gut microbiomes of these two groups.

The study design is shown as **Fig. 1,** and the characteristics of the CHC patients and healthy individuals are presented in **Table 1** (cross-sectional study) **and Tables S2-S3** (longitudinal study).

Gut microbiome analysis

Total bacterial DNA was isolated from stool samples using the bead-beating method, followed by phenol extraction [1]. The variable V1-V2 (27F-354R primers) or V3-V4 (341F-785R primers) regions of the 16S ribosomal RNA gene were amplified by polymerase chain reaction in the cross-sectional and longitudinal study, respectively, and then subjected to high-throughput sequencing using the MiSeq paired-end sequencing system (Illumina Inc., San Diego, CA, USA) [1]. The sequences obtained were processed using the QIIME2 platform (qiime2-2023.2, https://qiime2.org) [4]. Briefly, the paired-end sequences were merged, trimmed, denoised, and clustered into amplicon sequence variants (ASVs). The representative sequence of each ASV was taxonomically classified using the classify-sklearn program with the SILVA 138 database (https://www.arb-silva.de/). The statistics, ASV, and taxonomy tables in the 16S rRNA amplicon profiling are summarized in the **Tables S4-S8**.

For assessing the α-diversity of the gut microbiome in CHC patients during HCV infection, post-SVR at various clinical stages, and in healthy individuals, a rarefaction curve of the Shannon-Wiener index was constructed to reflect the ASV composition in each sample and increasing subsampling depth, in the QIIME2 platform. After the sampling depth was rarefied at 7,500 reads in each group, a significant difference of the Shannon-Wiener index in each group to the healthy group was examined by the nonparametric Dunnett's test in the R nparcomp package.

The β-diversity of gut microbiome was estimated using the Bray-Curtis and Jaccard distances, calculated based on the ASV composition of each sample. Biases in the alpha diversity between groups were calculated by the Permanova analysis in 'adonis' function in the R vegan package. The contribution of host factors to the ordination was calculated using the 'envfit' function in the R vegan package.

The genus composition of each sample was averaged within each group and examined by nonparametric Dunnett's test.

The statistical significance of the change in the longitudinal measure to compare the gut microbiome in CHC patients during HCV infection and after SVR was examined using the Wilcoxon signed-rank test in the Stata program (SE12.0). Correlation of the change in the abundance of each genus and ASV with the liver function parameters was examined by the generalized estimating equations (GEE) in the Stata program. In the GEE analysis, the relative abundance of each genus or ASV was normalized across all samples, each liver function parameter was converted to a Z-score, and applied for the GEE analysis with an unstructured correlation matrix and gaussian family speculation.

Measurement of the concentration of BAs

Of the 272 samples whose gut microbiota were analyzed, BA analysis was conducted on 176 patients whose sample volumes were sufficient for this purpose. The major 15 BAs in fecal samples from the 176 subjects (23 healthy individuals, 58 CH-HCV, 42 LC/HCC-HCV, 16 CH-SVR, 37 LC/HCC-SVR) were quantified using high-performance liquid chromatography-triple quadrupole mass spectrometry (Shimadzu, LCMS 8050) with nor-deoxycholic acid as an internal standard, as described previously [2]. The subjects included 100 CHC patients with HCV infection and 23 healthy individuals who were enrolled in our previous study [2] and their BA data were combined into this study. The relative abundance data of the 15 BAs in each sample was subjected to between-group statistical difference analysis, as well as the cluster analysis.

For cluster analysis, Euclidean distances between samples were calculated based on the BA composition data of 176 samples by the ‘vegdist’ function in the R vegan package. Based on the distance, the distribution of samples was displayed in the principal coordinate analysis (PCoA) and were then subjected to the pam clustering in the R cluster package, resulting in three clusters with the highest Calinski-Harabasz index and reasonable Silhouette width (0.47), calculated by the R clusterSim package.

The three clusters were defined by the most abundant BA, namely DCA, LCA, and UDCA types. The distribution of the three BA types was compared statistically between healthy individuals and CHC patient groups or between before and after SVR, among healthy individuals and CHC patients during HCV infection and after SVR at various clinical stages by Macnemar's test in the Stata program. The correlation of host factors to the BA-based ordination was analyzed by the envfit function in the R vegan package and displayed as vector arrows on the PCoA plot, in addition to the estimates of r^2 and probability "p".

To find genera statistically abundant or deficient in a certain BA type, the relative abundance of each genus in samples was compared by the pairwise Wilcoxon rank-sum test with adjustment by Benjamin-Hochberg procedure.

Analysis of whole-transcriptome sequencing (RNA-seq)

The procedure has been described in our previous paper [2]. We randomly enrolled 65 Japanese, biopsy-proven CHC patients with HCV infection, including mild CHC (F0–2, n = 22) and advanced CHC (F3–4, n = 43). Additionally, 28 CHC patients after SVR, including mild CHC (F0-2, n = 12) and advanced CHC (F3-4, n = 16), were enrolled in this study. The data were obtained from the International Cancer Genome Consortium (ICGC). Our HCC patients were also included in the project. For healthy liver controls, 12 patients with similar ages to the CHC patients were enrolled. These samples were surgical resections from organ donors. RNA-Seq data were collected from Sequence Read Archive. Statistical analysis was performed using the Kruskal-Wallis test. Subsequently, multiple comparisons were conducted using the Steel-Dwass test, followed by Bonferroni adjustment. Clinical and pathological data are summarized in **Table S9**.

Statistics

In all of the statistical analysis, a *p* value <0.05 was considered statistically significant. The specific statistical methods applied are detailed in the corresponding subjections. For selected analyses, post hoc power calculations were conducted using G*Power version 3.1.9.2 to obtain 1-β (power). For the GEE analysis, power (1-β) was manually calculated by using the estimated effect size (delta), residual standard deviation, and intra-subject correlation (rho), as GPower dose not support GEE models.

Study approval

Written informed consent was obtained from each individual and the study was approved by each institutional ethics committee, in accordance with the Declaration of Helsinki. The methods were carried out in accordance with the approved guidelines.

Accession numbers of pyrosequencing data

The raw sequence data have been deposited in the DNA Data Bank of Japan (DDBJ) sequence read archive (DRR584747-DRR584826 and DRR585283-DRR585554) under BioProject no. PRJDB18460 (PSUB023519) and PRJDB18519 (PSUB023615).

Supplementary figures

(A)

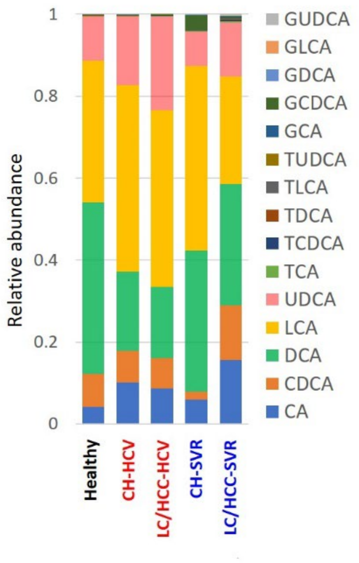

(B)

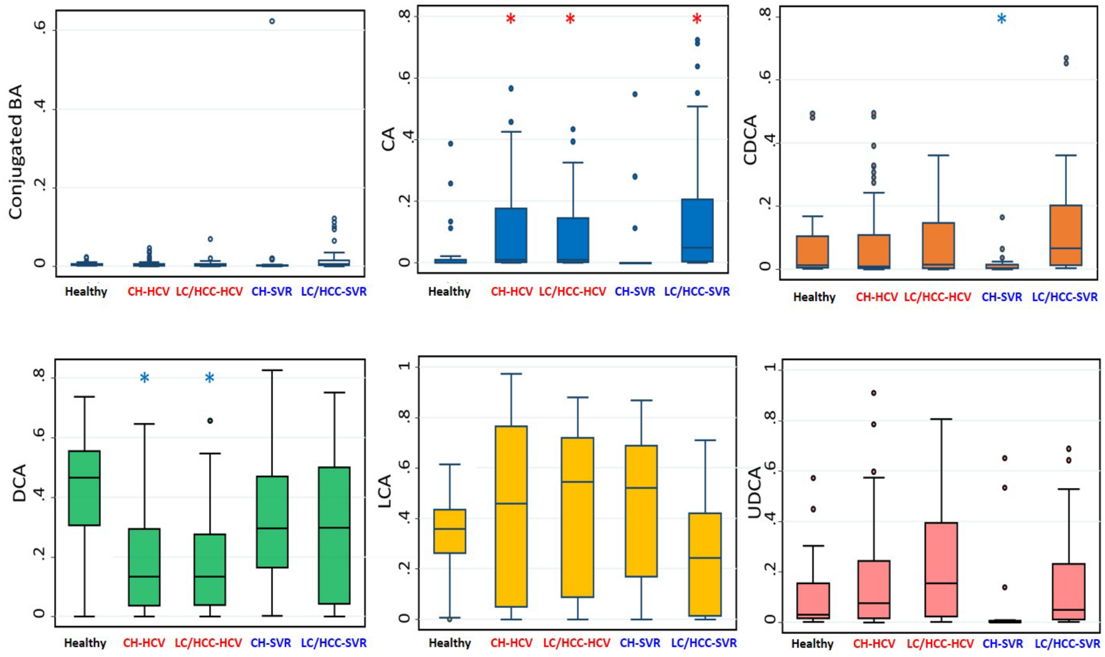

Fig. S1: Fecal BA profiles obtained from CHC patients and healthy individuals

(A) Stacked bar graph showing the relative BA composition in the fecal samples, averaged within each group. **(B)** Box plot showing the distribution of relative abundance of each BA in feces from each subject group. Red and blue stars above the

bars represent statistically significant higher or lower values than the healthy control group, respectively ($p < 0.05$ in Steel test).

Abbreviations: CHC, chronic hepatitis C; BA, bile acid; HCV, hepatitis C virus; CH, chronic hepatitis; LC, cirrhosis; HCC, hepatocellular carcinoma in cirrhosis; SVR, sustained virological response; CH-HCV group, persistently normal alanine aminotransferase (PNALT) or CH under HCV infection group; LC/HCC-HCV group, LC or HCC under HCV infection group; CH-SVR group, PNALT or CH after SVR group; LC/HCC-SVR group, LC or HCC after SVR group; healthy group, healthy individuals group; ASV, amplicon sequence variants; CA, cholic acid; CDCA, chenodeoxycholic acid; DCA, deoxycholic acid; LCA, lithocholic acid; UDCA, ursodeoxycholic acid; TCA, taurocholic acid; TCDCA, taurochenodeoxycholic acid; TDCA, taurodeoxycholic acid; TLCA, taurolithocholic acid; TUDCA, tauroursodeoxycholic acid; GCA, glycocholic acid; GCDCA, glycochenodeoxycholic acid; GDCA, glycodeoxycholic acid; GLCA, glycolithocholic acid; GUDCA, glycoursodeoxycholic acid.

(A)

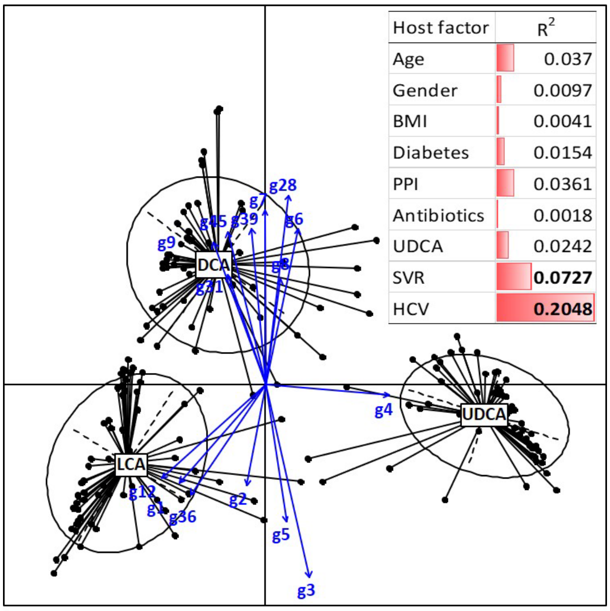

(B)

GeneID	Genus	CA	CDCA	DCA	LCA	UDCA
g1	*Bacteroides*	-0.085	-0.097	0.016	0.132	-0.028
g2	*Bifidobacterium*	-0.042	-0.110	-0.053	0.090	-0.055
g3	*Streptococcus*	0.114	0.027	**-0.250**	0.008	**0.149**
g4	*[Ruminococcus]_gnavus_group*	0.034	0.017	-0.057	0.035	-0.009
g5	*Anaerostipes*	-0.040	0.026	-0.038	-0.006	0.044
g6	*Prevotella_9*	0.001	0.010	0.086	-0.059	-0.108
g7	*Agathobacter*	-0.053	0.021	**0.205**	-0.046	-0.114
g8	*Blautia*	-0.005	0.082	0.087	-0.146	-0.003
g9	*Megamonas*	-0.045	-0.026	**0.168**	0.035	-0.101
g12	*Ligilactobacillus*	-0.042	-0.080	0.074	0.038	-0.114
g28	*Roseburia*	-0.053	0.020	0.144	-0.040	-0.081
g31	*Ruminococcus*	-0.012	0.001	0.039	-0.028	0.016
g36	*Escherichia-Shigella*	0.001	0.006	0.008	0.099	-0.029
g39	*Faecalibacterium*	-0.121	-0.037	**0.206**	-0.097	-0.064
g45	*Lachnoclostridium*	-0.091	-0.081	**0.239**	-0.002	**-0.165**

Fig. S2: Correlation of the abundance of each genus and host factors to the fecal BA ordination

(A) Correlation of the abundance of each genus and host factors to the principal coordination of subject' BA profile. The inlet table indicates the coefficient of determination (r) of each host factor to the BA principal coordination. Bold letters indicate the correlation to be significant ($p < 0.05$). Blue arrows indicate the correlation of each genus (gX, most 20 abundant genera listed in **(B)**) to the BA principal coordination ($p < 0.4$).

(B) Spearman correlation of the relative abundance of each genus to the relative abundance of each BA. Bold letters indicate the correlation with significance (*p* <0.05).
Abbreviations: BA, bile acid; PCA, principal component analysis; BMI, body mass index; PPI, proton pump inhibitor; UDCA, ursodeoxycholic acid; CHC, chronic hepatitis C; CH, chronic hepatitis; LC, cirrhosis; HCC, hepatocellular carcinoma in cirrhosis; SVR, sustained virological response; chenodeoxycholic acid; DCA, deoxycholic acid; LCA, lithocholic acid.

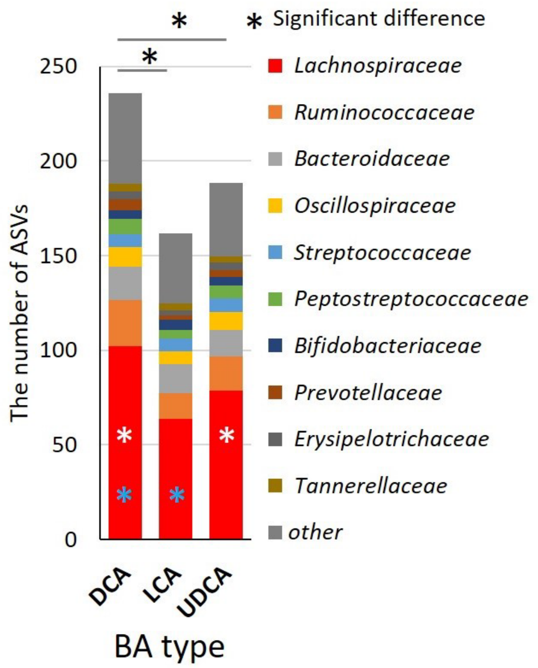

Fig. S3: Comparison of observed ASV numbers among different BA type groups
Stacked bar graph showing the number of ASVs observed in each BA type subject. Asterisks inside the bar indicate a significant difference in the number of ASVs, corresponding to family Lachnospiraceae, across 3 BA types. Asterisks over the bar indicate a significant difference of total number of ASVs observed in each BA type. The data were averaged within each BA type.

Abbreviations: ASV, amplicon sequence variant; BA, bile acid; chenodeoxycholic acid; DCA, deoxycholic acid; LCA, lithocholic acid; UDCA, ursodeoxycholic acid.

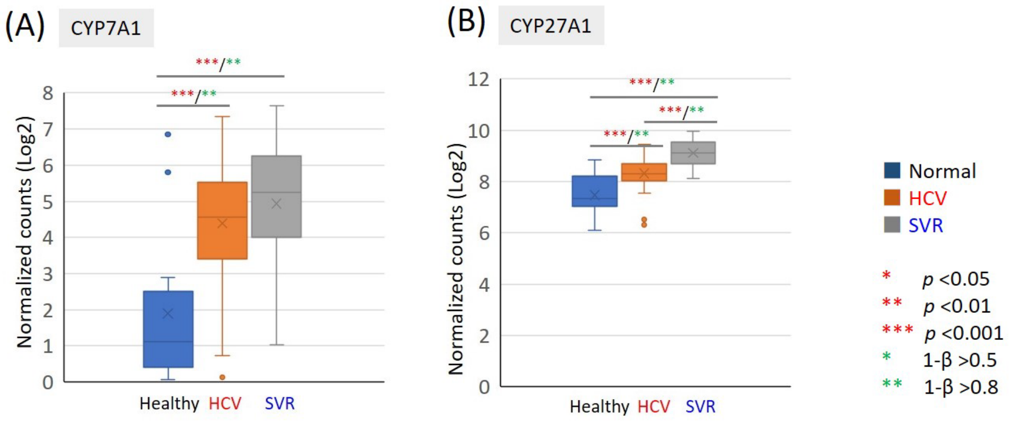

Fig. S4. Changes in the gene expression levels of enzymes involved in BA biosynthesis, in the liver after SVR

Expression in the liver of genes involved in the metabolism of BAs in 65 CHC patients (F0-2 [n = 22] and F3-4 [n = 43]) and individuals with healthy livers (n = 12) by RNA-Seq. Statistical analysis was performed by Kruskal-Wallis multiple comparison. *P*-values were adjusted using the Benjamini-Hochberg method. Red and green asterisks show the significant differences between the groups and the statistical power, respectively. (**A**) CYP7A1, (**B**) CYP27A1.

Abbreviations: BA, bile acid; SVR, sustained virological response; CHC, chronic hepatitis C; RNA-Seq, transcriptional analysis; Healthy, individuals with healthy liver; CYP7A1, cholesterol 7α-hydroxylase; CYP27A1, sterol 27-hydroxylase.

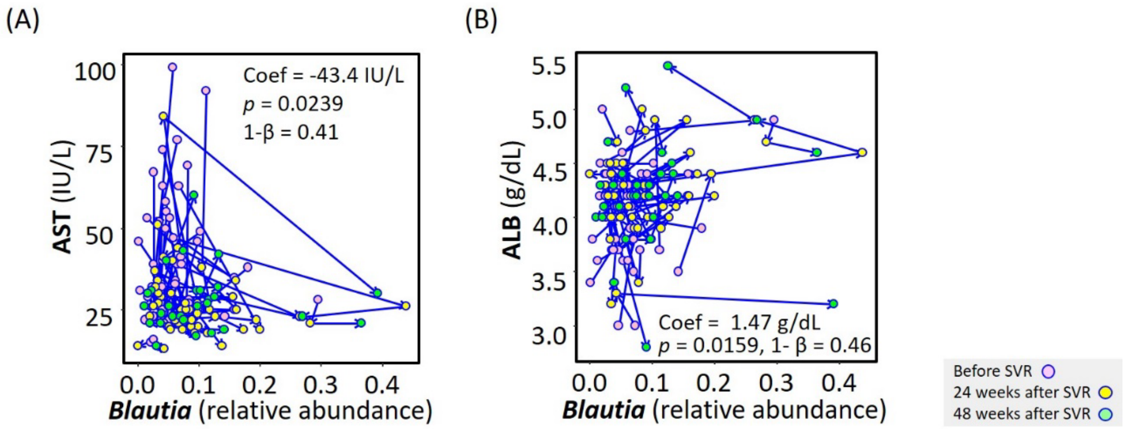

Fig. S5. Longitudinal changes of the gut microbiome after SVR and their association with liver inflammation indicators

Longitudinal changes in the abundance of *Blautia*, (**A**) AST and (**B**) ALB. Pink, yellow, and green dots represent data during HCV infection, SVR24 and SVR48, respectively. Arrow lines connect data from the same individuals over time.

Abbreviations: SVR, sustained virological response; ALT, alanine-2-oxoglutarate aminotransferase; AST, asparate-2-oxoglutarate aminotransferase; ALB, serum albumin; HCV, hepatitis C virus; SVR24, the status of 24 weeks after SVR; SVR48, the status of 48 weeks after SVR.

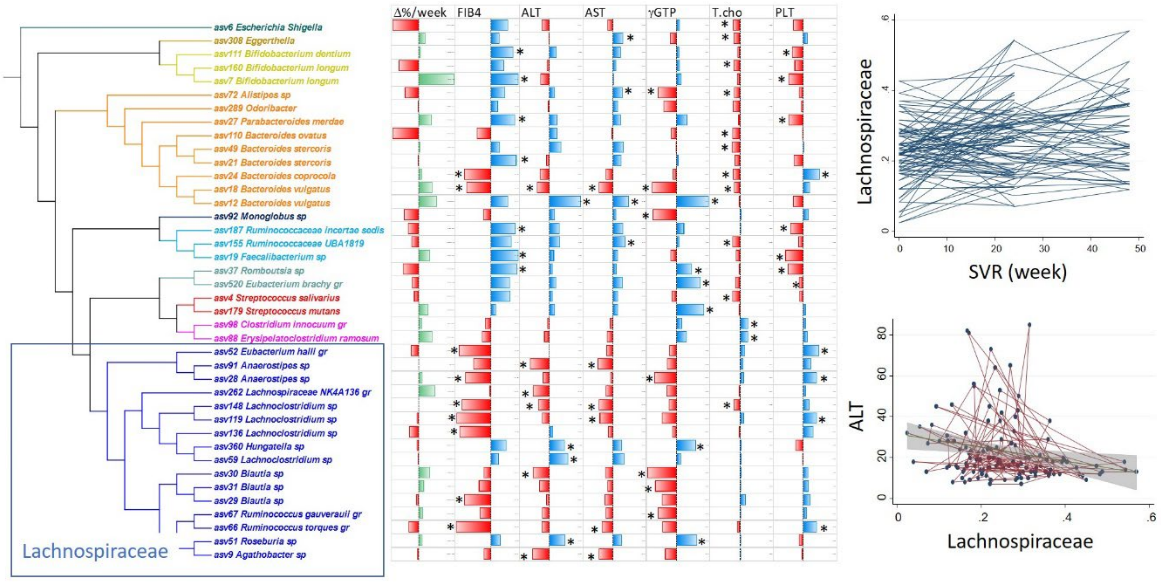

Fig. S6. Correlation with the abundance of each ASV to the liver function

The correlation between the change in abundance of each ASV and the alteration in liver function indicators before and after HCV elimination is shown in the bar graph besides the phylogenetic tree of each ASV. In the horizontal bar graphs, the column to the right of each ASV name illustrates the occupancy percentage change of each ASV, before and after achieving SVR. Green indicates an increase and red a decrease in ASVs (%). The subsequent columns to the right demonstrate the relationship between liver function indicators and each ASV. Red indicates an inverse correlation, while blue signifies a direct correlation. The ASVs named in dark blue letters belong to the order *Lachnospirales.* Statistically significant correlations are indicated by asterisks (in the GEE analysis, *p <0.05). The upper right line graph indicates the change in the total abundance of ASV belonging to order *Lachnospirales* over time post-SVR. The lower right line graph shows the correlation between the change in the ALT levels and change in the abundance of *Lachnospirales.* Each dot represents ALT and the abundance of *Lachnospirales* in each sample.

Abbreviations: SVR, sustained virological response; FIB4, fibrosis-4 index; ALT, alanine-2-oxoglutarate aminotransferase; AST, asparate-2-oxoglutarate aminotransferase; γGTP, γ-glutamyl transpeptidase; t-cho, total cholesterol; PLT, platelet count.

Supplementary tables

Patients profile		Previous report (n = 166) [1]	This study (n = 249)	
Under HCV infection	PNALT	18	18	CH-HCV group (n = 95)
	CH	84	77	
	LC	40	51	LC/HCC-HCV group (n = 79)
	HCC	24	28	
	Total	166	174	
After SVR	PNALT		4	CH-SVR group (n = 29)
	CH		25	
	LC		29	LC/HCC-SVR group (n = 46)
	HCC		17	
	Total		75	

Table S1: CHC patients enrolled in cross-sectional study

Abbreviations: CHC, chronic hepatitis C; HCV, hepatitis C virus; CH, chronic hepatitis; LC, cirrhosis; HCC, hepatocellular carcinoma in cirrhosis; SVR, sustained virological response; CH-HCV group, persistently normal alanine aminotransferase (PNALT) or CH under HCV infection group; LC/HCC-HCV group, LC or HCC under HCV infection group; CH-SVR group, PNALT or CH after SVR group; LC/HCC-SVR group, LC or HCC after SVR group; healthy group, healthy individuals group.

Enrolled patients		Numbers of provided samples		
		Under HCV infection	24 weeks after SVR	48 weeks after SVR
PNALT (n = 4)	CH group (n = 29)	29	29	18
CH (n = 25)				
LC (n =19)	LC/HCC group (n = 20)	20	20	11
HCC (n = 1)				
Total	49	49	49	29

Table S2: CHC patients enrolled in longitudinal study

Abbreviations: CHC, chronic hepatitis C; HCV, hepatitis C virus; CH, chronic hepatitis; LC, cirrhosis; HCC, hepatocellular carcinoma in cirrhosis; SVR, sustained virological response; CH-HCV group, persistently normal alanine aminotransferase (PNALT) or CH under HCV infection group; LC/HCC-HCV group, LC or HCC under HCV infection group; CH-SVR group, PNALT or CH after SVR group; LC/HCC-SVR group, LC or HCC after SVR group; healthy group, healthy individuals group.

Characteristics	Category (Number of candidates)		*p* values
	CH group (n = 29)	LC/HCC group (n = 20)	
Gender (M/F）	9/20	7/13	0.77
Age (years)	66.1 ± 12.6	72.7 ± 5.9	0.057
PLT (× 104/mm3)	20.4 ± 5.7	11.8 ± 5.7	< 0.0001
PT (%)	94.2 ± 9.5	84.8 ± 14.8	0.012
Alb (g/dL)	4.1 ± 0.4	4.0 ± 0.4	0.2
AST (IU/L)	37.3 ± 22.3	44.9 ± 13.0	0.18
ALT (IU/L)	30.1 ± 20.4	32.3 ± 11.9	0.68
γGT (IU/L)	22.3 ± 10.3	56.1 ± 57.5	0.0032
T-Bil (mg/dL)	1.1 ± 1.2	0.9 ± 0.4	0.58
AFP (ng/ml)	4.8 ± 7.2	11 ± 8.9	0.01
PIVKA-II (mAU/ml)	20.7 ± 13.6	23.2 ± 11	0.5
FIB-4 index	2.4 ± 1.2	6.3 ± 3.6	< 0.0001

Table S3: Demographics and clinical characteristics of CHC patients (n = 49) for longitudinal study

Continuous data are expressed as means ± standard deviation. Bold letters indicate a significant difference in one-way ANOVA followed by Tukey–Kramer post analysis.

Abbreviations: CHC, chronic hepatitis C; CH, persistently normal alanine aminotransferase or chronic hepatitis; LC/HCC, cirrhosis or hepatocellular carcinoma; healthy, healthy individuals; M, male; F, female; PLT, platelet count; PT, prothrombin time; Alb, serum albumin; AST, asparate-2-oxoglutarate aminotransferase; ALT, alanine-2-oxoglutarate aminotransferase; γGT, γ-glutamyl transpeptidase; T-Bil, total bilirubin; AFP, alpha fetoprotein; PIVKA-II, protein induced by vitamin K absence or antagonist-II; FIB-4 index, fibrosis-4 index.

**Cross-sectional Study (n = 272)**	input paired seqs.	quality pass merged seqs.	ASV	Genus	Family	Phylum
total	14,548,385	10,751,608	12,424	331	104	13
mean per sample	53,487	39,528	180.5	54.0	25.1	5.4
std per sample	52,455	40,981	126.8	23.7	7.5	1.1
max per sample	245,711	190,575	714	132	49	9
min per sample	11,674	7,740	19	12	10	3

**Longitudinal Study (n = 149)**	input paired seqs.	quality pass merged seqs.	ASV	Genus	Family	Phylum
total	15,272,826	6,936,502	3,846	333	95	15
mean per sample	120,258	54,618	149.6	66.65354	28.92913	5.9
std per sample	35,878	19,231	59.6	24.79451	8.14178	1.3
max per sample	234,529	107,499	331	127	53	10
min per sample	32,228	4,795	48	21	13	3

Table S4: Statistics in 16S rRNA profiling in this study

Abbreviations: ASV, amplicon sequence variants.

Table S5: ASV table in the cross-sectional study

Table S6: Taxonomy table in the cross-sectional study

Table S7: Taxonomy table in the longitudinal study

Table S8: Taxonomy table in the longitudinal study

Due to the large size of **Tables S5-S8**, they are provided separately as an Excel file. Please refer to the attached file for detailed data.

Characteristics at the time of liver biopsy	Category (Number of candidates)		
	CHC under HCV infection (n = 65)	CHC after SVR (n = 28)	Healthy (n = 12)
Gender (M/F)	44/21		9/3
Age (years)	$71.8 \pm 7.0^{a, b}$	$65.4 \pm 6.3^{a, c}$	$54.6 \pm 14.0^{b, c}$
Fibrosis stage (F0/F1/F2/F3/F4)	2/8/12/12/31	0/6/6/8/8	n.d.

Table S9: Characteristics of CHC patients (n = 64) and individuals with healthy livers (n = 12) whose liver tissues were used for RNA-Seq

Age data are expressed as mean ± standard deviation. Superscript letters indicate a significant difference in one-way ANOVA followed by Tukey-Kramer post-hoc analysis (p <0.0001: a, b and c).

Abbreviations: CHC, chronic hepatitis C; RNA-Seq, transcriptional analysis; HCV, hepatitis C virus; SVR, sustained virological response; Healthy, individuals with healthy liver; M, male; F, female; n.d., not determined.

	Dim1	Dim2	r2	Pr(>r)	
Age	0.08518	0.99637	0.0074	0.411	
Gender	0.79319	0.60897	0.0156	0.178	
BMI	-0.6382	0.76987	0.0017	0.846	
Diabetes	0.69724	0.71684	0.0124	0.24	
PPI	0.58862	0.80841	0.0534	0.003	**
Antibiotics	-0.1046	0.99451	0.0024	0.755	
H2.blocker	0.67999	0.73322	0.0256	0.044	*
UDCA	0.91945	-0.3932	0.0189	0.131	
Beta.blocker	-0.59632	-0.80275	0.0133	0.213	
SVR	-0.56379	-0.82592	0.5976	0.001	***
ClinicalStage			0.3091	0.001	***
CH-HCV	0.0622	0.0577	0.3091		
LC/HCC-HCV	0.0302	0.069	0.3091		
CH-SVR	-0.0997	-0.1557	0.3091		
LC/HCC-SVR	-0.0971	-0.0788	0.3091		

Table S10: Correlation of hosts and possible confounding factors with the PCoA ordination based on Bray-curtis distance across ASV composition of the 272 samples

Abbreviations: PCoA, principal coordinate analysis, ASV, amplicon sequence variants; Dim, Dimension; BMI, body mass index; PPI, proton pump inhibitor; H2 blocker, histamine H2-receptor; UDCA, ursodeoxycholic acid; SVR, sustained virological response; CH-HCV, persistently normal alanine aminotransferase (PNALT) or CH under HCV infection group; LC/HCC-HCV, LC or HCC under HCV infection group; CH-SVR, PNALT or CH after SVR group; LC/HCC-SVR, LC or HCC after SVR group.

	Dim1	Dim2	r2	Pr(>r)	
Age	0.07387	0.99727	0.0074	0.419	
Gender	0.7165	0.69759	0.0141	0.191	
BMI	-0.7349	0.67818	0.0017	0.833	
Diabetes	0.63166	0.77524	0.0121	0.23	
PPI	0.54035	0.84144	0.0524	0.005	**
Antibiotics	-0.19762	0.98028	0.0028	0.724	
H2.blocker	0.61152	0.79123	0.0251	0.055	.
UDCA	0.95311	-0.30264	0.0189	0.072	.
Beta.blocker	-0.47819	-0.87826	0.0147	0.185	
SVR	-0.47747	-0.87865	0.5933	0.001	***
ClinicalStage			0.3004	0.001	***
CH-HCV	0.0438	0.05			
LC/HCC-HCV	0.0194	0.056			
CH-SVR	-0.0655	-0.128			
LC/HCC-SVR	-0.0687	-0.0691			

Table S11: Correlation of hosts and possible confounding factors with the PCoA ordination based on Jaccard distance across ASV composition of the 272 samples

Abbreviations: PCoA, principal coordinate analysis, ASV, amplicon sequence variants; Dim, dimension; BMI, body mass index; PPI, proton pump inhibitor; H2 blocker, histamine H2-receptor; UDCA, ursodeoxycholic acid; SVR, sustained virological response; CH-HCV, persistently normal alanine aminotransferase (PNALT) or CH under HCV infection group; LC/HCC-HCV, LC or HCC under HCV infection group; CH-SVR, PNALT or CH after SVR group; LC/HCC-SVR, LC or HCC after SVR group.

Supplementary references

[1] Inoue T, Nakayama J, Moriya K, et al. Gut Dysbiosis Associated With Hepatitis C Virus Infection. Clin Infect Dis 2018;67:869-877.

[2] Inoue T, Funatsu Y, Ohnishi M, et al. Bile acid dysmetabolism in the gut-microbiota-liver axis under hepatitis C virus infection. Liver Int 2022;42:124-134.

[3] Faul F, Erdfelder E, Buchner A, et al. Statistical power analyses using G*Power 3.1: tests for correlation and regression analyses. Behav Res Methods 2009;41:1149-1160.

[4] Bolyen E, Rideout JR, Dillon MR, et al. Reproducible, interactive, scalable and extensible microbiome data science using QIIME 2. Nat Biotechnol 2019;37:852-857.
