# Supplementary material for: Restoration of the gut-microbiota-liver axis after hepatitis C virus eradication
Source: JHEP Rep. 2025 Jun 24;7(9):101494. doi: 10.1016/j.jhepr.2025.101494 (PMC12355056; doi:10.1016/j.jhepr.2025.101494)
Supplement: Multimedia component 5 [file mmc5.pdf]

Research article

JHEP Reports

Restoration of the gut-microbiota-liver axis after hepatitis C virus eradication

Authors

Takako Inoue, Jiro Nakayama, Hiroshi Mori, …, Atsushi Toyoda, Ken Kurokawa, Yasuhito Tanaka

Correspondence

ytanaka@kumamoto-u.ac.jp (Y. Tanaka).

Graphical abstract

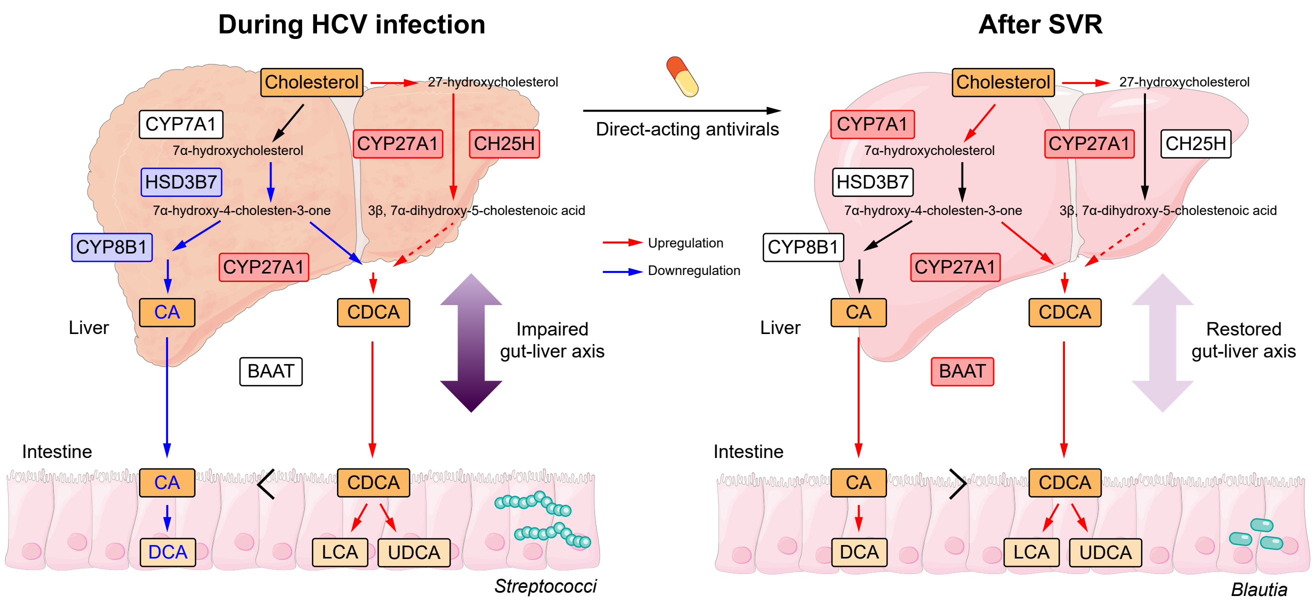

Highlights:

- This study investigates the gut-microbiota-liver axis in patients with chronic hepatitis C following SVR.
- During HCV infection, the classical bile acid biosynthetic pathway is suppressed.
- Intestinal deoxycholic acid levels are restored to normal following the achievement of SVR.
- An improvement in liver fibrosis and function is associated with an increase in *Blautia* species.

Impact and implications:

This study offers significant insights into the gut-microbiota-liver axis in patients with chronic hepatitis C following a sustained virological response. The findings demonstrate that HCV eradication promotes partial restoration of the dysbiotic gut microbiota, particularly an increase in the beneficial genus *Blautia*, as well as a rebalancing of the fecal bile acid profile. These changes are closely associated with significant improvements in liver fibrosis and function, highlighting a potential role of the gut microbiota in liver recovery and regeneration.

https://doi.org/10.1016/j.jhepr.2025.101494
© 2025 The Authors. Published by Elsevier B.V. on behalf of European Association for the Study of the Liver (EASL). This is an open access article under the CC BY license (http://creativecommons.org/licenses/by/4.0/). JHEP Reports, 2025, 7, 1–10

Restoration of the gut-microbiota-liver axis after hepatitis C virus eradication

Takako Inoue[1,†], **Jiro Nakayama**[2,†], Hiroshi Mori[3], Masaru Tanaka[2,4], Daisuke Nakagawa[2], Masaya Ohnishi[5,6], Yui Funatsu[2], Kei Moriya[7], Hideto Kawaratani[7], Hisayoshi Watanabe[8], Goki Suda[9], Yasuteru Kondo[10,11,17], Tatsuya Ide[12], Satoru Kakizaki[13], Satoshi Miuma[14], Atsushi Suetsugu[5], Kazuhito Kawata[15], Takao Watanabe[16], Etsuko Iio[17], Rie Momoda[2], Yutaka Suzuki[6], Akira Sakamaki[18], Tsunamasa Watanabe[19], Takehisa Watanabe[17], Katsuya Nagaoka[17], Yoichi Hiasa[16], Shuji Terai[18], Hitoshi Yoshiji[7], Atsushi Toyoda[3], Ken Kurokawa[3], Yasuhito Tanaka[17,*]

JHEP Reports **2025**. vol. 7 | 1–10

Check for updates

Background & Aims: We previously reported altered intestinal environmental features during HCV infection. Here, we aimed to characterize the gut-microbiota-liver axis in patients with chronic hepatitis C after a sustained virological response (SVR).

Methods: A total of 174 patients with HCV infection were enrolled in a cross-sectional study: 95 with chronic hepatitis (CH-HCV group) and 79 with cirrhosis or hepatocellular carcinoma (LC/HCC-HCV group). In addition, 75 post-SVR patients were included (CH-SVR group, n = 29; LC/HCC-SVR group, n = 46), along with 23 healthy individuals. A longitudinal study was subsequently conducted on 49 patients (CH, n = 29; LC/HCC, n = 20) with SVR at 24 and 48 weeks after the end of treatment. RNA sequencing was performed on 65 patients with HCV infection, 28 post-SVR patients, and 12 healthy controls.

Results: In the cross-sectional analysis, HCV eradication was associated with partial restoration of the dysbiotic gut microbiota, including reduced streptococcal overgrowth and an increase in the potentially beneficial genus *Blautia*, approaching levels seen in healthy individuals. Additionally, the aberrant fecal bile acid profile showed rebalancing, accompanied by restored expression of genes involved in the classical pathway of cholic and chenodeoxycholic acid biosynthesis. In the longitudinal study, improvements in liver fibrosis and function – evidenced by decreased Fibrosis-4 index and alanine aminotransferase levels – were significantly correlated with increased abundance of Blautia (p <0.0001 and p = 0.0344, respectively), suggesting a beneficial role in liver recovery.

Conclusion: The gut-microbiota-liver axis is partially restored following HCV eradication, with recovery from liver damage associated with the resurgence of commensal Lachnospiraceae species.

© 2025 The Authors. Published by Elsevier B.V. on behalf of European Association for the Study of the Liver (EASL). This is an open access article under the CC BY license (http://creativecommons.org/licenses/by/4.0/).

Introduction

Hepatitis C virus (HCV) infects approximately 58 million people worldwide, causing progressive liver damage that can lead to cirrhosis (LC) and hepatocellular carcinoma (HCC).[1,2] The advent of direct-acting antivirals (DAAs) has transformed HCV treatment, achieving sustained virological responses (SVR) in over 90% of patients.[3] This virological cure reduces the risks of hepatic and extrahepatic complications while improving health-related quality of life.[3] However, the post-SVR pathophysiological changes, particularly involving the gut-microbiota-liver axis, remain poorly understood.

We have reported that chronic hepatitis C (CHC) is associated with gut dysbiosis, marked by reduced bacterial diversity and compositional shifts, including decreased *Clostridiales* and increased *Streptococci* and *Lactobacilli*.[4] Additionally, we found HCV infection disrupts bile acid (BA) metabolism through an imbalance between the classical and alternative *de novo* synthesis pathways in the liver. This imbalance not only impairs hepatic function but also alters the gut microbial community, affecting the conversion of primary to secondary BAs. These changes are reflected in reduced fecal deoxycholic acid (DCA) to lithocholic acid (LCA) and ursodeoxycholic acid (UDCA) ratios, as well as decreased hepatic transcription of cytochrome P450 8B1 (CYP8B1), a critical enzyme in cholic acid synthesis. Similar disruptions have been observed in HCV-infected animal models, reinforcing the systemic effects of HCV on the gut-liver axis.[5]

BAs serve as both metabolic regulators and microbial modulators, making them critical components of the gut-microbiota-liver axis.[6] Through receptors such as FXR (farnesoid X receptor) and TGR5 (Takeda G-protein-coupled receptor 5), BAs regulate inflammation, glucose homeostasis, and lipid metabolism, while acting as antimicrobial agents to shape gut microbiota.[7] Dysregulated BA metabolism has been

* Corresponding author. Address: Department of Gastroenterology and Hepatology, Faculty of Life Sciences, Kumamoto University, 1-1-1 Honjo, Chuo-ku, Kumamoto, 860-8556, Japan; Tel.: +81-96-373-5146, fax: +81-96-371-0582.
E-mail address: ytanaka@kumamoto-u.ac.jp (Y. Tanaka).
† Equal contribution
https://doi.org/10.1016/j.jhepr.2025.101494

implicated in liver disease progression, emphasizing its importance in understanding CHC pathophysiology and recovery post-SVR.[5,7,8]

Regarding the gut microbiome in CHC, while some studies have reported improvements in gut microbiota composition post-SVR, including increased short-chain fatty acid-producing bacteria such as *Blautia* and *Bifidobacterium*,[9,10] others observed limited or no changes, particularly in patients with advanced liver disease.[11,12] These discrepant findings suggest a complicated underlying interaction between the gut microbiota and the liver after SVR.

To address this subject, we conducted a longitudinal observational study, following the cross-sectional investigation, to investigate the recovery of the gut microbiota in conjunction with liver function. Notably, we focused on BA metabolism in the gut microbial community and bile acid biosynthesis in the liver, to gain new insights into the dynamic interaction within the gut-microbiota-liver axis.

Patients and methods

The study design and methods are detailed in the supplementary materials. This retrospective study included 272 participants: 174 patients with active HCV infection, 75 post-SVR patients, and 23 healthy individuals. Among the patients with HCV, 166 had been previously reported,[4] and eight new cases were added in this study.

Patients with persistently normal alanine aminotransferase (ALT) values (PNALT) and chronic hepatitis (CH) were classified as CH-HCV, while those with LC and HCC were grouped as LC/HCC-HCV. Similarly, post-SVR patients were categorized into CH-SVR (PNALT and CH) and LC/HCC-SVR (LC and HCC). We analyzed gut microbiome and BA composition across these four groups and healthy individuals.

Additionally, 49 CHC patients from the cross-sectional study were included in a longitudinal analysis to assess gut microbiome changes at 24 and 48 weeks post-SVR (SVR24 and SVR48, respectively). Details are provided in Fig. 1 and Table S1; patient characteristics are summarized in Table 1 (cross-sectional) and Table S2–S3 (longitudinal). Definitions of clinical stages and inclusion/exclusion criteria were described in our previous papers.[4,5] All patients diagnosed with HCC had a background of LC.

The procedures for gut microbiome analysis, including DNA extraction from stool samples, 16S rRNA gene amplification, high-throughput sequencing, and data processing using QIIME2 (qiime2-2023.2, https://qiime2.org),[13] are detailed in the supplementary material. Summary statistics for 16S rRNA profiling, amplicon sequence variants (ASVs), and taxonomic classifications are provided in Table S4–S8. α- and β-diversity indices of gut microbiota across clinical stages (pre- and post-SVR) and comparisons with healthy individuals are described in the supplementary material. Additionally,

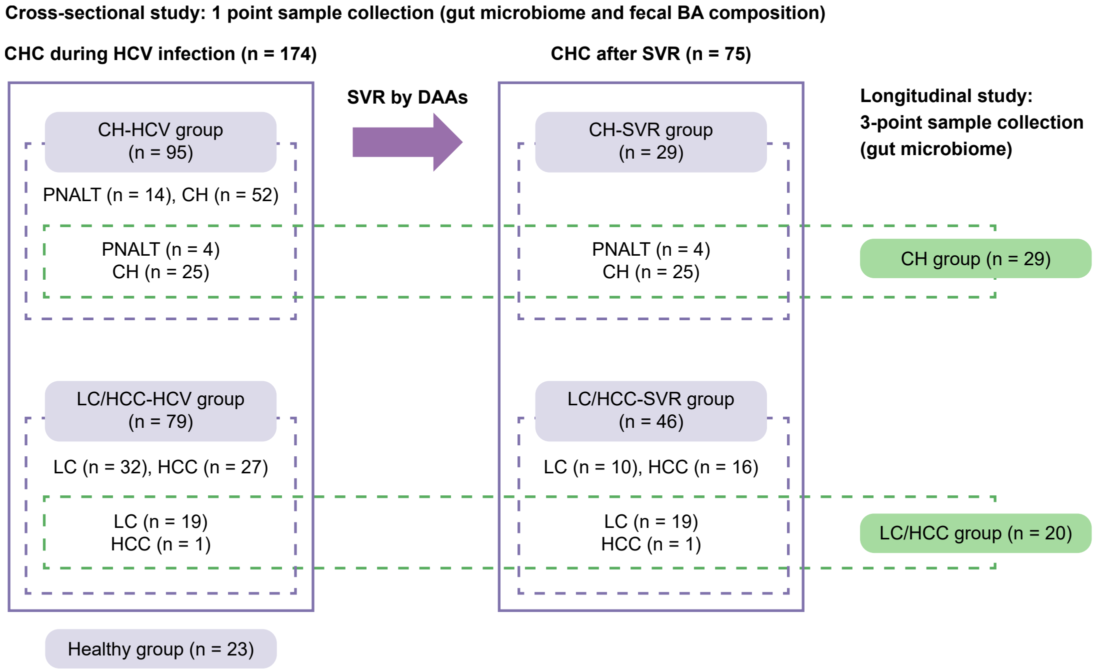

Fig. 1. Study design of the cross-sectional and longitudinal studies. In the cross-sectional study, a one-point sample was collected from each individual and examined for gut microbiome, fecal BA composition, and gene transcription. Five groups (CH-HCV, LC/HCC-HCV, CH-SVR, LC/HCC-SVR, and healthy groups) were examined. In the longitudinal study, 3-point samples (during HCV infection, SVR24, and SVR48) were collected from each individual and the gut microbiome was examined. Three groups (CH, LC/HCC, and healthy groups) were examined. All cases analyzed in the longitudinal study were also enrolled in the cross-sectional study. BA, bile acid; CH, chronic hepatitis; CH-HCV group, PNALT or CH under HCV infection group; CH-SVR group, PNALT or CH after SVR group; CHC, chronic hepatitis C; HCC, hepatocellular carcinoma in cirrhosis; healthy group, healthy individuals group; LC, cirrhosis; LC/HCC-HCV group, LC or HCC under HCV infection group; LC/HCC-SVR group, LC or HCC after SVR group; PNALT, persistently normal alanine aminotransferase; SVR, sustained virological response.

Table 1. Demographics and clinical characteristics of patients during HCV infection (n = 174), after SVR (n = 75), and of healthy individuals (n = 23) in the cross-sectional study.

Characteristics	Category (number of candidates)				Healthy group (n = 23)
	During HCV infection		After SVR		
	CH-HCV group (n = 95)	LC/HCC-HCV group (n = 79)	CH-SVR group (n = 29)	LC/HCC-SVR group (n = 46)	
Gender (M/F)	40/55	39/40	13/16	21/25	15/8
Age (years)	66.3 ± 12.9[a]	71.5 ± 9.3[a,b]	65.2 ± 12.0	70.7 ± 9.1[c]	61.3 ± 8.1[b,c]
PLT (×10^4/mm^3)	18.3 ± 5.1[d,e,f]	10.2 ± 44[d,g]	20.3 ± 5.1[e,g,h]	11.6 ± 4.1[f,h]	n.d.
PT (%)	91.2 ± 12.3[i]	78.3 ± 14.9[i,j,k]	99.4 ± 7.4[j]	90.0 ± 13.1[k]	n.d.
ALB (g/dl)	4.1 ± 0.4[l,m]	3.6 ± 0.6[l,n,o]	4.4 ± 0.3[m,n]	4.2 ± 0.5[o]	n.d.
AST (IU/L)	44.4 ± 41.5[p]	53.9 ± 43.2[q,r]	23.4 ± 8.8[p,q]	31.6 ± 19.8[r]	n.d.
ALT (IU/L)	49.7 ± 107.4	38.4 ± 38.4	16.8 ± 9.5	21.4 ± 13.3	n.d.
GGT (IU/L)	33.5 ± 31.9	45.5 ± 42.1	19.0 ± 7.5[s]	58.7 ± 127.5[s]	n.d.
T-Bil (mg/dl)	0.9 ± 0.7	1.3 ± 1.1	0.9 ± 0.7	1.2 ± 1.3	n.d.
AFP (ng/ml)	6.4 ± 14.5	113.5 ± 702.5	3.2 ± 1.8	9.3 ± 20.5	n.d.
PIVKA-II (mAU/ml)	20.7 ± 14.3	2,145.9 ± 17,360.7	30.8 ± 58.4	109.0 ± 502.9	n.d.
FIB-4 index	3.3 ± 2.2[t,u]	7.4 ± 4.1[t,v,x]	2.0 ± 1.0[v,y]	5.1 ± 3.2[u,x,y]	n.d.

AFP, alpha fetoprotein; ALB, serum albumin; ALT, alanine aminotransferase; AST, aspartate aminotransferase; FIB-4 index, fibrosis-4 index; GGT, γ-glutamyltransferase; healthy, healthy individuals; n.d., not determined; PIVKA-II, protein induced by vitamin K absence or antagonist-II; PLT, platelet count; PT, prothrombin time; T-Bil, total bilirubin.
Continuous data are expressed as means ± SD. Superscript letters indicate a significant difference in one-way ANOVA followed by Tukey–Kramer post analysis (p <0.001: e, f, g, h, j, k, l, m, p, u, v, w and x; p <0.01: d; p <0.05: a, b, c, l, n, o, q, r, s and t). There is no significant difference in sex ratio between each CHC group and the healthy group.

longitudinal microbiome changes post-SVR and their association with liver function were analyzed at the genus level in CH and LC/HCC groups, as detailed in the supplementary material.

Among the 272 individuals analyzed for gut microbiota, BA analysis was performed on 176 patients – including 100 with active HCV infection, 53 post-SVR, and 23 healthy individuals – with sufficient sample volumes. Fifteen major fecal BAs were quantified using high-performance liquid chromatography coupled with triple quadrupole mass spectrometry (LCMS-8050, SHIMADZU CORPORATION), as previously described.[5] Details of fecal BA profile comparisons across clinical stages and healthy individuals are provided in the supplementary material.

Whole-transcriptome sequencing (RNA-seq) procedures have been previously described.[5] We enrolled 65 patients with HCV infection, 28 post-SVR patients, and 12 age-matched healthy liver controls from organ donors. RNA-seq data were obtained from the Sequence Read Archive, and clinical and pathological data are summarized in Table S9.

Details on statistics and accession numbers for the 16S rRNA gene sequence data are provided in the supplementary material. All participants provided written informed consent, and the study was approved by institutional ethics committees in accordance with the Declaration of Helsinki.

Results

Clinical characteristics of patients with active HCV and healthy individuals

In the cross-sectional study, 32 of the LC/HCC-HCV group (40.5%) had decompensated cirrhosis. Advanced investigations beyond ultrasonography included biopsy (n = 4), MRI (n = 19), CT (n = 12), elastography plus MRI (n = 7), MRI plus CT (n = 16), and MRI plus biopsy (n = 21). Demographic and clinical differences among groups are summarized in Table 1, with no significant age or sex differences between CH-HCV, CH-SVR, and healthy groups.

In the longitudinal study, 49 patients with CHC (29 CH group and 20 LC/HCC group) were enrolled. Sample size calculations confirmed sufficient statistical power to monitor *Blautia* abundance changes. The minimum required sample sizes – 28 for CH and 15 for LC/HCC – were determined based on mean and variance in pre- and post-SVR groups (details in the supplementary materials). Significant demographic and clinical differences are shown in Table S3.

Cross-sectional findings

Recovery of gut microbial diversity (α-diversity) within individuals after SVR

The Shannon–Wiener index, which is a measure of taxonomic richness and evenness within an individual, was lower in the CH-HCV and LC/HCC-HCV groups than the healthy group, suggesting an imbalanced microbiome damaged during HCV infection, so-called dysbiosis. After SVR, α-diversity recovered to a level similar to the healthy group, which was more obvious in the CH-SVR than the LC/HCC-SVR group, suggesting the liver damage is even worse in the LC/HCC groups (Fig. 2A,B).

Recovery of the gut microbial community after SVR

Gut microbial community structure was compared among patients with CHC at different clinical stages and healthy individuals using Bray-Curtis and Jaccard distances, calculated from ASV composition data. Principal coordinate analysis based on these matrices showed distinct clustering of patients with CHC and healthy individuals (Fig. 3A,B). Jaccard distance indicated post-SVR samples clustered with healthy individuals, suggesting gut microbiome recovery after HCV eradication (Fig. 3B).

Streptococcus salivarius, known to overgrow in association with HCV infection, was identified as a characteristic microbiome component in patients with CHC (Figs. 3A,B). Other physiological factors – diabetes, obesity, and antibiotic use – showed weaker correlations with microbiome composition

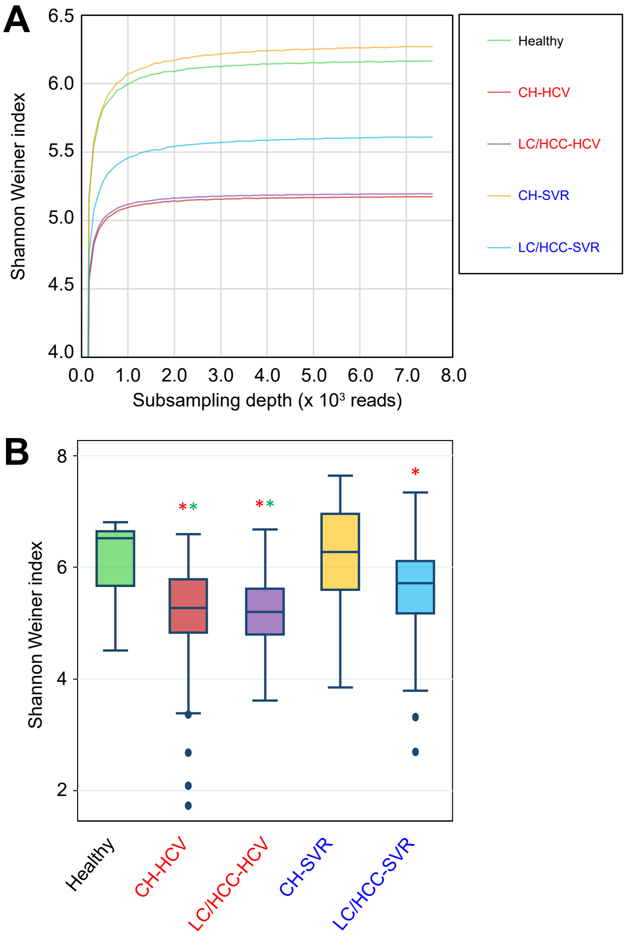

Fig. 2. α-diversity of the gut microbiome of patients during HCV infection and after SVR. (A) Rarefaction curve of the Shannon–Wiener index with increasing sampling depth. (B) Box plot showing Shannon–Wiener indices for each group at a sampling depth of 7,500 reads, including the minimum, maximum, quartiles, median, and outliers. Statistical differences from the healthy control were assessed using non-parametric Dunnett's test. Red and green asterisks indicate significant differences (p <0.05) with sufficient power (1-β >0.8). CH, chronic hepatitis; CH-HCV, PNALT or CH under HCV infection group; CH-SVR, PNALT or CH after SVR group; CHC, chronic hepatitis C; HCC, hepatocellular carcinoma in cirrhosis; healthy, healthy individuals group; LC, cirrhosis; LC/HCC-HCV, LC or HCC under HCV infection group; LC/HCC-SVR, LC or HCC after SVR group; PNALT, persistently normal alanine aminotransferase; SVR, sustained virological response.

(Fig. 3A,B). No participants reported alcohol consumption or selective eating habits. Proton pump inhibitor administration, as reported,[14,15] influenced gut microbiome structure but had a minor impact compared to HCV eradication. These findings indicate that microbiome alterations were primarily driven by HCV infection and eradication, with minimal confounding effects (Fig. 3A,B; Table S10 and S11).

Changes in gut microbiome during HCV infection and after its eradication

Genus compositions of fecal samples from patients with CHC at various clinical stages were compared to those of healthy individuals (Fig. 4). There were three genus groups, classified according to their changing patterns, as follows: Type A comprises genera which decreased during HCV infection but did not recover after HCV elimination. The most dominant genus, *Bacteroides*, and *Faecalibacterium*, known as a beneficial butyrate producer, belong to this type. Type B comprises genera which decreased during HCV infection but recovered after HCV eradication. *Blautia*, *Fusicatenibacter*, and *Roseburia*, fall into this category. The post-SVR recovery of these genera was more complete in the CH group than the LC/HCC group. Type C comprises genera which increased with HCV infection. *Streptococcus* and *Bifidobacterium* belong to this type. The increase in these two genera was more evident in the LC/HCC group than the CH group. *Streptococcus* tended to decrease after HCV eradication, while the level of *Bifidobacterium* did not change after HCV eradication (Fig. 4).

Rebalance of fecal BA composition after HCV eradication

Fecal BA composition, including 15 BA molecules, was analyzed in 176 samples from patients with CHC (at different clinical stages) and healthy individuals. As in the previous study, DCA ratios decreased in CH and LC/HCC groups but partially recovered post-SVR (Fig. S1). While LCA and UDCA abundance showed no significant difference from healthy controls, their variance increased (Fig. S1). Clustering analysis grouped BA profiles into three types dominated by DCA, LCA, or UDCA (Fig. 5A, inset). Although total BA levels were higher in LCA and UDCA types, the difference was not statistically significant. Healthy individuals predominantly had the DCA type, whereas CH and LC/HCC groups showed higher proportions of LCA or UDCA (Fig. 5B), contributing to variance in LCA and UDCA levels (Fig. S1B). Post-SVR, BA profiles tended to shift toward DCA, especially in the LC/HCC group (Fig. 5A). Other physiological factors had no statistical impact on BA typing (Fig. S2A).

Few bacterial genera differed significantly among BA types; *Bacteroides* was lower, and *Lachnoclostridium* was higher in the DCA type (Fig. 5C). LCA and UDCA types showed fewer ASVs, particularly within *Lachnospiraceae* (Fig. S3). Correlation analysis revealed genera within *Clostridia*, including *Agathobacter*, *Megamonas*, *Faecalibacterium*, and *Lachnoclostridium*, positively associated with DCA, whereas *Streptococcus* showed a negative correlation (Fig. S2A). *Lachnoclostridium*, including *L. scindens* (a secondary BA producer), had a strong positive correlation with DCA. No genera showed significant correlation with LCA, although Blautia, which is classified as B-type, exhibited the strongest negative coefficient (Fig. 4).

Comparison of the expression of BA metabolism-related genes, before and after HCV eradication

This study analyzed gene expression levels of key enzymes involved in BA biosynthesis using RNA-seq data, comparing individuals with healthy livers, patients with HCV infection, and those post-SVR.

In the classical pathway, cholesterol 7-alpha-hydroxylase (CYP7A1), which converts cholesterol to 7α-hydroxycholesterol,[16] was upregulated in patients with CHC during HCV infection and remained elevated post-SVR, compared to individuals with healthy livers (Fig. S4A). Conversely, 3 beta-hydroxysteroid dehydrogenase type 7 (HSD3B7) and CYP8B1, downstream of CYP7A1, were downregulated during HCV infection but recovered post-SVR to levels similar to

A

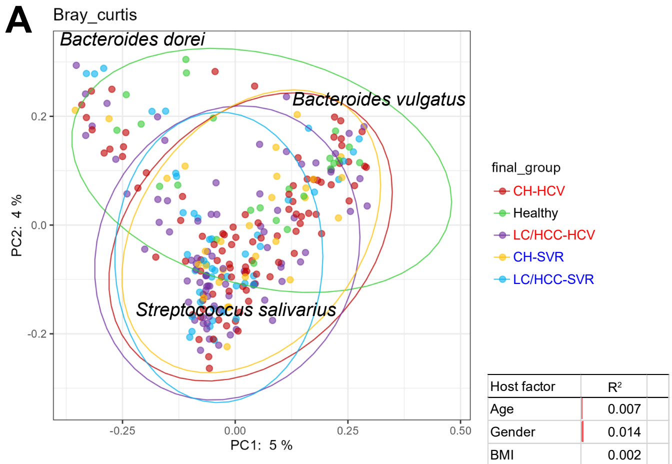

	Healthy	CH-HCV	LC/HCC-HCV	CH-SVR
CH-HCV	0.0005			
LC/HCC-HCV	0.0005	0.0107		
CH-SVR	0.0032	0.2068	0.0005	
LC/HCC-SVR	0.0005	0.0247	0.0964	0.1847

Host factor	R^2	
Age	0.007	
Gender	0.014	
BMI	0.002	
Diabetes	0.012	
PPI	0.052	*
Antibiotics	0.003	
UDCA	0.019	.
SVR treatment	0.593	**
Hepatitis stage	0.300	**

B

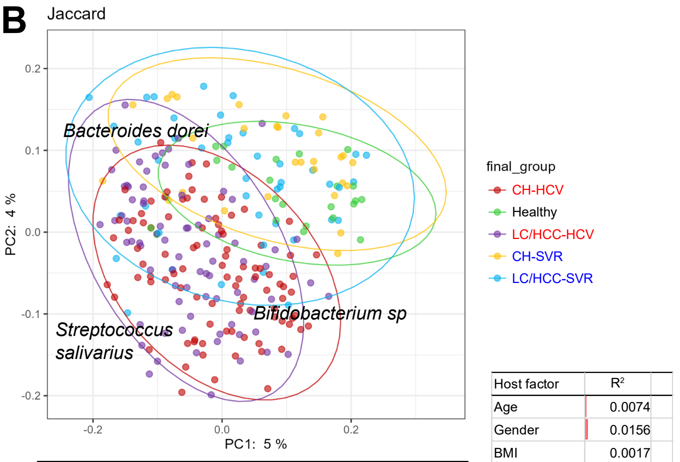

	Healthy	CH-HCV	LC/HCC-HCV	CH-SVR
CH-HCV	0.0001			
LC/HCC-HCV	0.0001	0.0003		
CH-SVR	0.0003	0.0001	0.0001	
LC/HCC-SVR	0.0001	0.0001	0.0001	0.0108

Host factor	R^2	
Age	0.0074	
Gender	0.0156	
BMI	0.0017	
Diabetes	0.0124	
PPI	0.0534	*
Antibiotics	0.0024	
UDCA	0.0189	
SVR treatment	0.5976	**
Hepatitis stage	0.3091	**

Fig. 3. β-diversity of the gut microbiome of patients during HCV infection and after SVR. PCoA plots showing the microbiome beta-diversity of samples from patients with CHC at different clinical stages and healthy individuals. Diversities were calculated using (A) Bray-Curtis and (B) Jaccard distances based on ASV composition dissimilarities. Ellipses indicate 95% confidence intervals for group distributions, with statistical differences shown below the plots (*p* values from Permanova analysis). Correlations of host factors to ordination are listed beside the plots as r^2 values, with single asterisks (p <0.05) and double asterisks (p <0.001) indicating significance. ASV, amplicon sequence variant; CH, chronic hepatitis; CH-HCV, PNALT or CH under HCV infection group; CH-SVR, PNALT or CH after SVR group; CHC, chronic hepatitis C; HCC, hepatocellular carcinoma in cirrhosis; healthy, healthy individuals group; LC, cirrhosis; LC/HCC-HCV, LC or HCC under HCV infection group; LC/HCC-SVR, LC or HCC after SVR group; NMDS, non-metric multidimensional scaling; PCoA, principal component analysis; PNALT, persistently normal alanine aminotransferase; SVR, sustained virological response.

those in individuals with healthy livers (Figs. 6A,B). Sterol 27-hydroxylase, downstream of CYP8B1, exhibited greater upregulation post-SVR compared to during HCV infection (Fig. S4B).

In the alternative pathway, cholesterol 25-hydroxylase was upregulated in patients during HCV infection but returned to levels similar to those of individuals with healthy livers after SVR (Fig. 6C). Small heterodimer partner, which mediates FXR signaling to CYP7A1,[17] was significantly upregulated in patients during HCV infection, and recovered after SVR to levels similar to the those in individuals with healthy livers (Fig. 6D). Fig. 6 illustrates that BA biosynthetic pathways toward DCA recover, whereas the pathway leading to LCA via chenodeoxycholic acid remains upregulated post-SVR.

All comparisons with p <0.05 exhibited $1 - \beta$ >0.8, confirming robust statistical power for this RNA-seq analysis. Fig. 6E presents a simplified BA metabolic pathway in patients during HCV infection and post-SVR.

Longitudinal changes of the gut microbiome before and after HCV eradication and their association with liver fibrosis and inflammatory indicators

In the CH group, *Blautia* and *Faecalibacterium* increased significantly at SVR24 and SVR48 (p <0.05), *Subdoligranulum* at SVR24 (p <0.05), and *Collinsella* at SVR48 (p <0.05). Meanwhile, *Streptococcus*, *[Eubacterium]_hallii_group*, and *[Ruminococcus]_torques_group* decreased significantly at SVR48 (each p <0.05). In the LC/HCC group, only *Blautia* increased significantly at SVR48 (p <0.05), with no significant changes in other species (Fig. 7A).

Fig. 7B illustrates correlations between bacterial abundance changes and liver function parameters. *Blautia* negatively correlated with the Fibrosis-4 index (FIB-4) (p <0.001, 1-ß = 0.91), ALT (p <0.05, 1-ß = 0.37), aspartate aminotransferase (AST) (p <0.05, 1-ß = 0.41), and positively with serum albumin (ALB) (p <0.05, 1-ß = 0.46), indicating its association with liver function recovery. *Agathobacter* showed negative correlations with ALT and AST (both p <0.05, 1-ß = 1.00), but not FIB-4, while *Streptococcus* negatively correlated only with ALB (p <0.05, 1-ß = 0.87). *Faecalibacterium* had a positive correlation with FIB-4 (p <0.05, 1-ß = 0.82), despite its post-SVR increase.

Fig. S6 presents ASV-level data, showing ASVs in *Lachnospiraceae* (including *Blautia* and *Lachnoclostridium*) correlated negatively with FIB-4, ALT, AST, and γ-glutamyltransferase. Individual-level changes in *Blautia* and its correlation with liver fibrosis and inflammation are depicted in Fig. 7C–E and Fig. S4. Post-SVR, *Blautia* increased most notably at 24 weeks in patients with initially lower levels (Fig. 7C–E and Fig. S5). Its increase strongly correlated with reductions in FIB-4 (coefficient = -0.081 per 1% increase), ALT (coefficient = -0.396 IU/L per 1% increase), AST (coefficient = -0.434 IU/L per 1% increase, Fig. S5A), and an ALB rise (coefficient = 0.0147 g/dl per 1% increase, Fig. S5B), indicating that improvements in liver fibrosis and inflammation are linked to the increase in *Blautia*.

Discussion

The cross-sectional observational study indicated that HCV eradication tended to lead to recovery of the gut microbiota from the dysbiotic state observed in patients with CHC. Although the microbiome structures of patients after SVR did

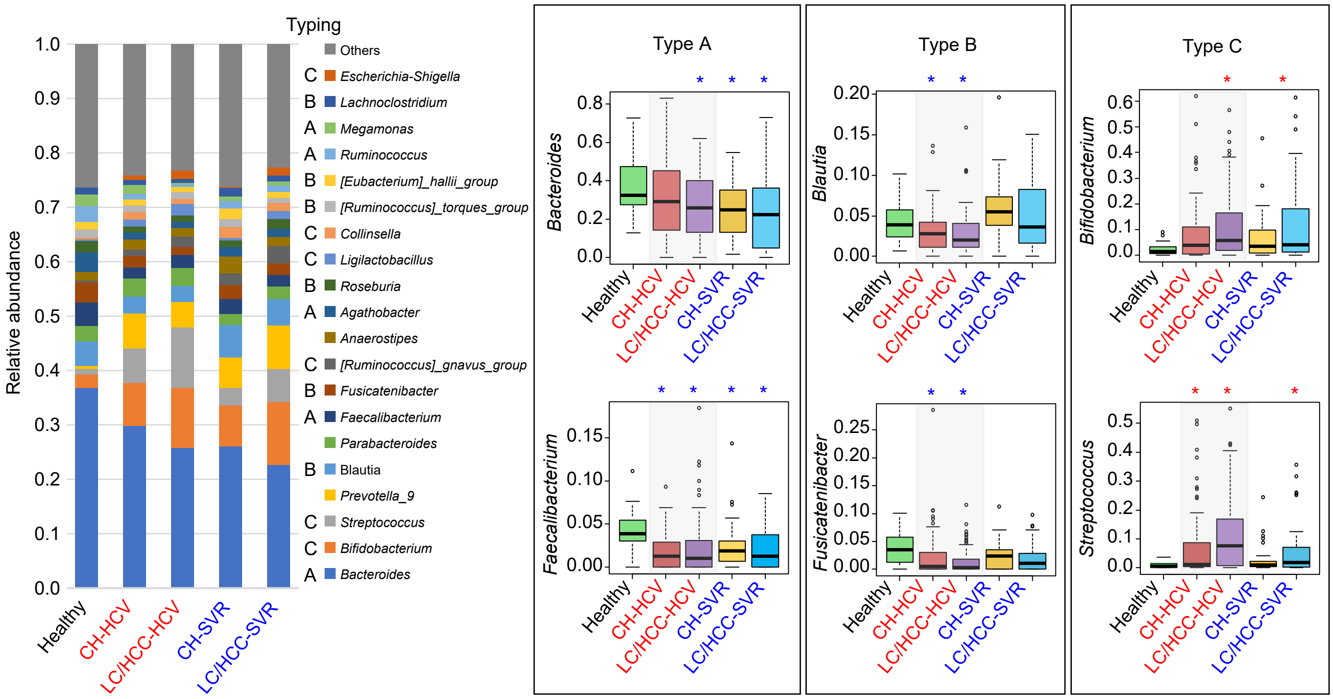

Fig. 4. Impact of HCV infection and eradication on gut microbiota. The genus composition of each sample was averaged within each group and displayed in the stacked bar chart. The relative abundance of each genus at each hepatitis stage was compared to the healthy group and categorized as follows: Type A decreased during HCV infection and did not recover after eradication. Type B decreased during HCV infection but tended to recover after eradication. Type C increased during HCV infection and showed a decreasing trend after eradication. Representative genera for each type are shown in the box plot, which includes minimum, maximum, quartiles, median, and outliers. Asterisks (*) indicate significant differences (p <0.05) from the healthy group (red: increase, blue: decrease) as determined by non-parametric Dunnett's test. CH, chronic hepatitis; CH-HCV, PNALT or CH under HCV infection group; CH-SVR, PNALT or CH after SVR group; CHC, chronic hepatitis C; HCC, hepatocellular carcinoma in cirrhosis; healthy, healthy individuals group; LC, cirrhosis; LC/HCC-HCV, LC or HCC under HCV infection group; LC/HCC-SVR, LC or HCC after SVR group; SVR, sustained virological response.

not fully overlap with those of the healthy group (Fig. 3), the reduced α-diversity, indicative of a dysbiotic state, had recovered to healthy levels in the CH group and partially recovered in the LC/HCC group (Fig. 2). The recovery pattern of gut bacteria depended on the genera (Fig. 4). *Blautia* and *Fusicatenibacter* recovered to levels statistically comparable to those of healthy controls in both the CH and LC/HCC groups, whereas the increase in *Bifidobacterium* and *Streptococcus* and the decrease in *Bacteroides* and *Faecalibacterium* did not normalize. These findings more or less align with several previous reports.[10–12,18]

The subsequent longitudinal analysis revealed an increase in potentially beneficial bacteria, such as *Faecalibacterium* and *Blautia*, after HCV eradication. While numerous studies have highlighted the positive effects of *Faecalibacterium* on host health,[19] this study identified *Blautia*, rather than *Faecalibacteria*, as a key commensal genus associated with improved liver function and reduced fibrosis. *Blautia* is notably abundant in the Japanese gut microbiome and plays pivotal roles in the health of the host.[20] For example, a Japanese cohort study demonstrated an inverse correlation between *Blautia* abundance and obesity and type 2 diabetes mellitus, while an animal study suggested that *Blautia* and its metabolites ameliorate these metabolic disorders through their anti-inflammatory properties and modulation of the gut environment and lipid metabolism.[21,22] The anti-inflammatory activity of *Blautia* may support the recovery of liver function and tissue repair, while the lower level of *Blautia* could compromise the liver defense mechanism in CHC. In addition to the microbiome analysis, we performed an intensive fecal metabolome analysis of BA composition, evaluated in conjunction with the liver tissue RNA-seq data analysis, to capture the status of *de novo* BA biosynthesis in the livers of patients with CHC. Our BA typing analysis revealed the shift from DCA type to LCA type in patients with HCV infection and the recovery to the DCA type after HCV eradication (Figs. 5 and 6).

RNA-seq analysis of the liver biopsy samples further demonstrated recovery of the balance between the classical pathway and the alternative pathway.[17] As shown in Fig. S2, a great number of Clostridia species, which are mostly commensal bacteria generally observed in healthy microbiota and found to be reduced in patients with HCV in our previous study,[4] correlated with the DCA type, while non-Clostridia group, including *Bacteroides*, *Bifidobacterium*, *Streptococcus*, and *Escherichia-Shigella*, inversely correlated with the DCA type. Because 7α-dehydroxylation probably occurs evenly on cholic acid and chenodeoxycholic acid through the activity of certain bacterial enzymes, it is thought that the shift of BA-type is caused by the alteration of liver function, as shown by the RNA-seq data. However, the finding that the total concentration of BAs in DCA-type feces is lower than in LCA/UDCA-type feces (inset in Fig. 5A) and that the number of ASVs observed in the DCA type is higher than in the LCA/UDCA type (Fig. S3) suggest the presence of antimicrobial

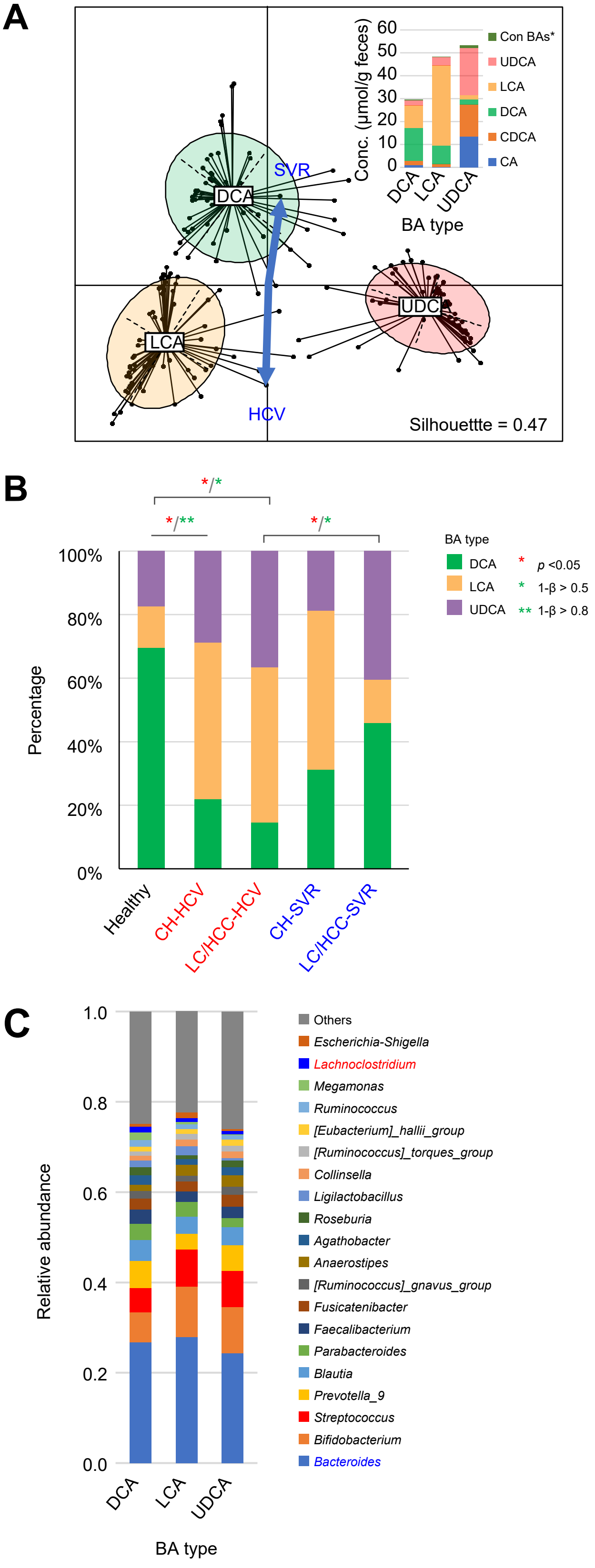

Fig. 5. Fecal BA typing of patients with CHC at different clinical stages and healthy individuals. (A) Ordination and clustering of samples based on fecal BA composition, defining three clusters: DCA (n = 57), LCA (n = 65), and UDCA (n = 54). Blue arrows represent host factors significantly correlated with the ordination. Euclidean distances were calculated from the relative abundances of 15 major BAs, and PAM clustering identified three clusters based on the highest Calinski-Harabasz index and a silhouette width of 0.47. An inset bar chart shows the BA composition for each type. (B) Distribution of healthy individuals and patients with CHC across the three BA types. Red asterisks denote significant group differences (p <0.05, McNemar's test). Single and double green asterisks indicate statistical power levels of 1-β <0.5 and 1-β <0.8, respectively. (C) Average genus composition within each BA type. Genera in red and blue indicate significantly higher and lower abundances in the DCA type compared to others (pairwise Wilcoxon rank-sum test with BH adjustment, p <0.05). ASV, amplicon sequence variants; BA, bile acid; CA, cholic acid; CDCA, chenodeoxycholic acid; CH, chronic hepatitis; CH-HCV, PNALT or CH under HCV infection group; CH-SVR, PNALT or CH after SVR group; CHC, chronic hepatitis C; DCA, deoxycholic acid; HCC, hepatocellular carcinoma in cirrhosis; healthy, healthy individuals group; LCA, lithocholic acid; LC, cirrhosis; LC/HCC-HCV, LC or HCC under HCV infection group; LC/HCC-SVR, LC or HCC after SVR group; SVR, sustained virological response; UDCA, ursodeoxycholic acid.

pressure, attributable to the high concentration of BAs in patients with CHC. Taken together, the changes in gut microbiota associated with shifts in BA type appear to result from the combined effects of both quantitative and qualitative changes in BAs.

In the case of MASLD (metabolic dysfunction-associated steatotic liver disease), a reduction of 7α-dehydroxylation leading to an increased ratio of primary to secondary BAs has been reported, in contrast to the imbalance between DCA and LCA found in this CHC study.[23–25] This is associated with gut microbiome dysbiosis, where the gut commensal population, including Clostridiales, decreases. Such dysbiosis is often observed in non-communicable diseases, suggesting a link between exposome factors, particularly unhealthy diet, and the exacerbation of the gut environment, such as an increase in bactericidal BA levels.[24] On the other hand, a study of patients infected with HBV indicated that a higher level of DCA was inversely associated with HCC risk,[25] in agreement with this study. The impact of the reduction of DCA caused by viral infection of liver tissues represents a different aspect of hepatitis progression, compared to hepatitis in non-communicable diseases.

Collectively, we propose the following model for the recovery of the liver-gut microbiome axis following SVR. HCV-damaged liver function leads to an imbalance in *de novo* BA biosynthesis, which recruits an LCA-type gut microbiome. This imbalance increases the level of highly hydrophobic and cytotoxic LCA and decreases the abundance of the commensal Clostridiales group, which has health-promoting effects (such as short-chain fatty acid production), exacerbating liver damage.[26] Following HCV eradication, the liver-gut microbiome axis begins to restore itself. *De novo* BA biosynthesis is rebalanced in the liver and the gut microbiota recovers from dysbiosis, thereafter improving liver function. The observed increase in *Blautia* levels during recovery is associated with reduced hepatic damage, suggesting a potential protective role in this context.

Our study has several limitations. First, the patients with CHC providing fecal samples and those providing liver tissues were not entirely the same, and RNA-seq data from patients with CHC and individuals with healthy livers were partially sourced from a database, limiting the feasibility of comprehensive correlation analyses. Second, in the longitudinal study, the sample size at SVR48 was limited because many patients

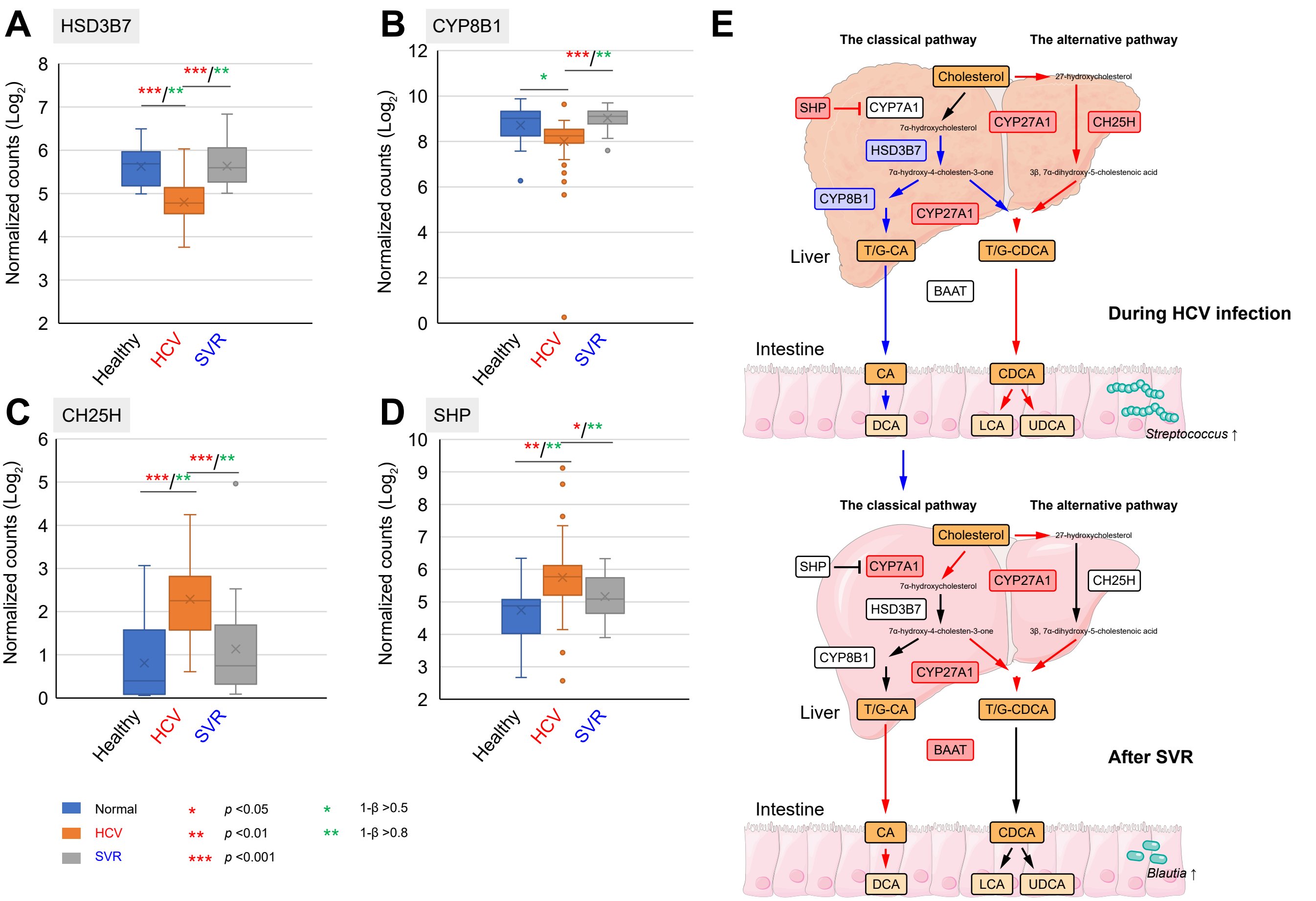

Fig. 6. Changes in the gene expression levels of enzymes involved in BA biosynthesis in the liver after SVR. Gene expression in the liver related to BA metabolism analyzed by RNA-seq in 65 patients with CHC (F0-2 [n = 22], F3-4 [n = 43]) and 12 healthy individuals. Red and green asterisks indicate significant differences and statistical power, respectively. (A) *HSD3B7*, (B) *CYP8B1*, (C) *CH25H,* and (D) *SHP.* (E) Comparison of BA dysmetabolism during HCV infection and after SVR. Red characters and arrows represent upregulated genes; blue denote downregulated genes. Statistical analysis was performed using the Kruskal-Wallis test, followed by Steel-Dwass test and Bonferroni adjustment. BA, bile acid; BAAT, bile acid-CoA amino acid N-acyltransferase; CA, cholic acid; CDCA, chenodeoxycholic acid; CH25H, cholesterol 25-hydroxylase; CHC, chronic hepatitis C; CYP27A1, sterol 27-hydroxylase; CYP7A1, cholesterol 7α-hydroxylase; CYP7B1, oxysterol 7α-hydroxylase; CYP8B1, cytochrome P450 8B1; DCA, deoxycholic acid; Healthy, individuals with healthy liver; HSD3B7, 3 beta-hydroxysteroid dehydrogenase type 7; LCA, lithocholic acid; RNA-Seq, transcriptional analysis; SHP, small heterodimer partner; SVR, sustained virological response; UDCA, ursodeoxycholic acid.

achieving SVR24 returned to their referring institutions. Third, we acknowledge that the use of separate cohorts for microbiome, BA, and hepatic transcriptomic analyses represents a significant limitation of this study, as it precludes direct cross-omic correlation and restricts the depth of mechanistic inference. This constraint limits our ability to derive integrative insights into gut-liver axis dynamics. Nevertheless, our findings provide valuable foundational knowledge, laying the groundwork for more comprehensive, multi-layered analyses in future research.

To refine future integrative analyses, we propose computational strategies to mitigate cohort separation effects. Bayesian hierarchical modeling and batch effect correction may help harmonize data from distinct biological sources, while emerging artificial intelligence-driven integration techniques offer promising approaches for aligning heterogeneous omic layers. Specifically, computational frameworks such as Multi-Omics Factor Analysis Plus (MOFA+) and Data Integration Analysis for Biomarker discovery using Latent cOmponents (DIABLO) are designed to infer cross-modal associations, even in partially matched or non-overlapping datasets. Implementing such approaches in future studies could enhance our ability to uncover mechanistic links between microbiome dynamics, BA metabolism, and hepatic gene expression. Despite these limitations, the proposed computational strategies offer a promising path toward bridging multi-omic gaps. To that end, we are actively planning prospective studies that incorporate harmonized sampling designs to enable fully integrative analyses.

In conclusion, HCV eradication corrects the imbalance of BA biosynthesis and allows the gut microbiome composition to recover, becoming closer to that of healthy individuals, with variations depending on the prior clinical stage of CHC. A key finding is the recovery of *Blautia* levels after HCV eradication, which is associated with improvements in liver fibrosis and inflammation markers. These results highlight

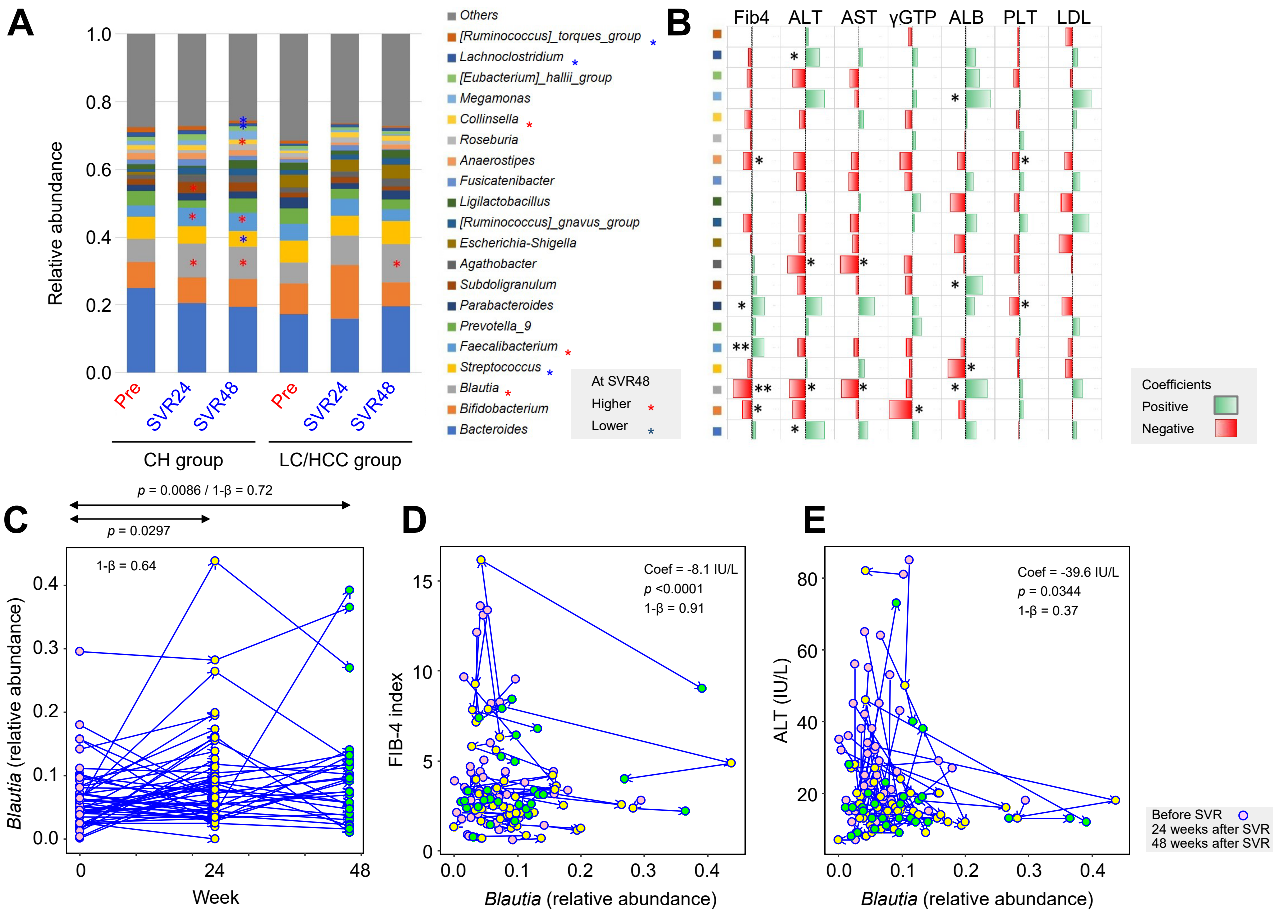

Fig. 7. Longitudinal changes of fecal microbiota before and after HCV eradication and their association with liver function indicators. (A) Genus-level gut microbiota changes after HCV elimination in CH and LC/HCC groups. Red and blue asterisks indicate significantly higher and lower levels compared to pre-eradication (Wilcoxon signed-rank test, $p < 0.05$). (B) Correlation of genus abundance changes with liver function indicators after HCV eradication. Green and red bars denote positive and negative coefficients, respectively. Statistically significant correlations are marked by asterisks (*$p < 0.05$, **$p < 0.001$, GEE analysis). Genera correspond to Fig. 7A. (C) Relative abundance of *Blautia* at SVR24 and SVR48. (D) Changes in FIB-4 index and *Blautia* abundance after SVR. (E) Changes in ALT levels and *Blautia* abundance after SVR. Pink, yellow, and green dots represent data before SVR, and at SVR24 and SVR48, respectively. Statistical power (1-β) is shown in the figure. ALB, serum albumin; ALT, alanine aminotransferase; AST, aspartate aminotransferase; CH, chronic hepatitis; CHC, chronic hepatitis C; FIB-4, fibrosis-4 index; GGT, gamma-glutamyltransferase; HCC, hepatocellular carcinoma; LDL, low density lipoprotein cholesterol; LC, cirrhosis; PNALT, persistently normal alanine aminotransferase; PLT, platelet count; Pre, the status of HCV infection; SVR, sustained virological response; SVR24, SVR at 24 weeks after treatment end; SVR48, SVR at 48 weeks after treatment end.

Blautia's potential as a live biotherapeutic product to aid the recovery of liver function alongside DAA therapy. Additionally, *Blautia* may serve as a biomarker for predicting liver fibrosis and inflammation outcomes, warranting further investigation in large-scale observational studies of DAA-treated patients. This study underscores the importance of targeting the gut microbiome to support liver recovery in patients with CHC.

Affiliations

[1]*Department of Clinical Laboratory Medicine, Nagoya City University Hospital, Nagoya, Japan;* [2]*Laboratory of Microbial Technology, Division of Systems Bioengineering, Department of Bioscience and Biotechnology, Faculty of Agriculture, Graduate School, Kyushu University, Fukuoka, Japan;* [3]*Advanced Genomics Center, National Institute of Genetics, Mishima, Japan;* [4]*JSR Corporation, Tokyo, Japan;* [5]*Department of Gastroenterology/Internal Medicine, Gifu University Graduate School of Medicine, Gifu, Japan;* [6]*Department of Computational Biology and Medical Sciences, Graduate School of Frontier Sciences, The University of Tokyo, Kashiwa, Japan;* [7]*Department of Gastroenterology and Hepatology, Nara Medical University, Kashihara, Japan;* [8]*Department of Gastroenterology and Hepatology, Tohoku Central Hospital of the Mutual Aid Association of Public School Teachers, Yamagata, Japan;* [9]*Department of Gastroenterology and Hepatology, Graduate School of Medicine, Hokkaido University, Sapporo, Japan;* [10]*Department of Hepatology, Sendai Kousei Hospital, Sendai, Japan;* [11]*Department of Hepatology, Sendai Tokushukai Hospital, Sendai Japan;* [12]*Division of Gastroenterology, Kurume University Medical Center, Kurume, Japan;* [13]*Department of Clinical Research, NHO Takasaki General Medical Center, Takasaki, Japan;* [14]*Department of Gastroenterology and Hepatology, Nagasaki University Graduate School of Biomedical Sciences, Nagasaki, Japan;* [15]*Hepatology Division, Department of Internal Medicine II, Hamamatsu University School of Medicine, Hamamatsu, Japan;* [16]*Department of Gastroenterology and Metabology, Ehime University Graduate School of Medicine, Toon, Japan;* [17]*Department of Gastroenterology and Hepatology, Faculty of Life Sciences, Kumamoto University, Kumamoto, Japan;* [18]*Division of Gastroenterology and Hepatology, Graduate School of Medical and Dental Sciences, Niigata University, Niigata, Japan;* [19]*Division of Gastroenterology and Hepatology, St. Marianna University School of Medicine, Kawasaki, Japan*

Abbreviations

ALB, serum albumin; ALT, alanine aminotransferase; ASVs, amplicon sequence variants; AST, aspartate aminotransferase; BA, bile acid; CH, chronic hepatitis; CH-HCV, PNALT or CH under HCV infection group; CH-SVR, PNALT or CH after SVR group; CHC, chronic hepatitis C; CYP7A1, cholesterol 7-alpha-hydroxylase; CYP8B1, cytochrome P450 8B1; DAAs, direct-acting antivirals; DCA, deoxycholic acid; FIB-4, Fibrosis-4 index; HCC, hepatocellular carcinoma; HSD3B7, 3 beta-hydroxysteroid dehydrogenase type 7; LC, cirrhosis; LC/HCC-HCV, LC or HCC under HCV infection group; LC/HCC-SVR, LC or HCC after SVR group; LCA, lithocholic acid; PNALT, persistently normal ALT values; RNA-seq, whole-transcriptome sequencing; SVR, sustained virological response; SVR24, SVR at 24 weeks after treatment end; SVR48, SVR at 48 weeks after treatment end; UDCA, ursodeoxycholic acid.

Financial support

This research was supported by AMED under Grant Number JP24fk0210103 and JP25fk0210172, the Ministry of Education, Culture, Sports, Science, and Technology (22K08037), and grant-in-aid for research from Nagoya City University.

Conflict of interest

Lecture Fees: AbbVie GK, Gilead Sciences, Inc. (Satoru Kakizaki), AbbVie GK, Gilead Sciences, Inc., Chugai Pharmaceutical Co., Ltd., ASKA Pharmaceutical Holdings Co., Ltd., OTSUKA Pharmaceutical Co., Ltd, Takeda Pharmaceutical Co., Ltd, GlaxoSmithKline PLC., AstraZeneca, Eisai, HU frontier (Yasuhito Tanaka). Consigned/Joint Research Expenses: Fujirebio, Inc., Sysmex Corporation (Takako Inoue and Yasuhito Tanaka), AbbVie GK., GlaxoSmithKline PLC., Gilead Sciences, Inc., Janssen Pharmaceutical K.K. (Yasuhito Tanaka). Scholarship donations: AbbVie GK., OTSUKA Pharmaceutical Co., Ltd (Yasuhito Tanaka).

Please refer to the accompanying ICMJE disclosure forms for further details.

Authors' contributions

Study concept and design (TI, JN, HM, AT, KK, YT), acquisition of samples (TI, JN, KM, HK, HW, GS, YK, TI, SK, SM, KK, TW, EI, RS, YH, TW, ST, HY), analysis and interpretation of data (TI, JN, HM, MT, DN, MO, YF, RM, YS), drafting of the manuscript (TI, YF, MO, JN), critical revision of the manuscript for important intellectual content (all authors).

Data availability

The data that support the findings of this study are available from the corresponding author upon reasonable request.

Acknowledgements

We thank the Center for Advanced Technical and Educational Supports, Faculty of Agriculture, Kyushu University for use of MiSeq and LCMS-8050. Additionally, we thank all participating patients and healthy individuals for providing samples and personal information.

Supplementary data

Supplementary data to this article can be found online at https://doi.org/10.1016/j.jhepr.2025.101494.

References

Author names in bold designate shared co-first authorship

[1] World Health Organization (WHO). Hepatitis C fectsheet. WHO web site; 2024. https://www.who.int/news-room/fact-sheets/detail/hepatitis-c:2024.
[2] Seeff LB. Natural history of chronic hepatitis C. Hepatology 2002;36:S35–S46.
[3] Ioannou GN, Feld JJ. What are the benefits of a sustained virologic response to direct-acting antiviral therapy for hepatitis C virus infection? Gastroenterology 2019;156:446–460 e442.
[4] Inoue T, Nakayama J, Moriya K, et al. Gut dysbiosis associated with hepatitis C virus infection. Clin Infect Dis 2018;67:869–877.
[5] Inoue T, Funatsu Y, Ohnishi M, et al. Bile acid dysmetabolism in the gut-microbiota-liver axis under hepatitis C virus infection. Liver Int 2022;42:124–134.
[6] Grüner N, Mattner J. Bile acids and microbiota: multifaceted and versatile regulators of the liver-gut Axis. Int J Mol Sci 2021;22:1397.
[7] Agus A, Clément K, Sokol H. Gut microbiota-derived metabolites as central regulators in metabolic disorders. Gut 2021;70:1174–1182.
[8] Farooqui N, Elhence A. Shalimar. A current understanding of bile acids in chronic liver disease. J Clin Exp Hepatol 2022;12:155–173.
[9] Ponziani FR, Putignani L, Paroni Sterbini F, et al. Influence of hepatitis C virus eradication with direct-acting antivirals on the gut microbiota in patients with cirrhosis. Aliment Pharmacol Ther 2018;48:1301–1311.
[10] **Chuaypen N**, **Jinato T**, Avihingsanon A, et al. Long-term benefit of DAAs on gut dysbiosis and microbial translocation in HCV-infected patients with and without HIV coinfection. Sci Rep 2023;13:14413.
[11] Huang PY, Chen CH, Tsai MJ, et al. Effects of direct anti-viral agents on the gut microbiota in patients with chronic hepatitis C. J Formos Med Assoc 2023;122:157–163.
[12] Wellhöner F, Döscher N, Woelfl F, et al. Eradication of chronic HCV infection: improvement of dysbiosis only in patients without liver cirrhosis. Hepatology 2021;74:72–82.
[13] Bolyen E, Rideout JR, Dillon MR, et al. Reproducible, interactive, scalable and extensible microbiome data science using QIIME 2. Nat Biotechnol 2019;37:852–857.
[14] Imhann F, Bonder MJ, Vich Vila A, et al. Proton pump inhibitors affect the gut microbiome. Gut 2016;65:740–748.
[15] Jackson MA, Goodrich JK, Maxan ME, et al. Proton pump inhibitors alter the composition of the gut microbiota. Gut 2016;65:749–756.
[16] Chiang JYL, Ferrell JM. Bile acid metabolism in liver pathobiology. Gene Expr 2018;18:71–87.
[17] Kwong E, Li Y, Hylemon PB, et al. Bile acids and sphingosine-1-phosphate receptor 2 in hepatic lipid metabolism. Acta pharmaceutica Sinica B 2015;5:151–157.
[18] Pérez-Matute P, Íñiguez M, Villanueva-Millán MJ, et al. Short-term effects of direct-acting antiviral agents on inflammation and gut microbiota in hepatitis C-infected patients. Eur J Intern Med 2019;67:47–58.
[19] Martín R, Rios-Covian D, Huillet E, et al. *Faecalibacterium*: a bacterial genus with promising human health applications. FEMS Microbiol Rev 2023;47:fuad039.
[20] Nishijima S, Suda W, Oshima K, et al. The gut microbiome of healthy Japanese and its microbial and functional uniqueness. DNA Res 2016;23:125–133.
[21] Hosomi K, Saito M, Park J, et al. Oral administration of *Blautia wexlerae* ameliorates obesity and type 2 diabetes via metabolic remodeling of the gut microbiota. Nat Commun 2022;13:4477.
[22] Konstanti P, Gómez-Martínez C, Muralidharan J, et al. Faecal microbiota composition and impulsivity in a cohort of older adults with metabolic syndrome. Sci Rep 2024;14:28075.
[23] **Mouzaki M**, **Wang AY**, Bandsma R, et al. Bile acids and dysbiosis in non-alcoholic fatty liver disease. PLoS One 2016;11:e0151829.
[24] **Montanari C**, **Parolisi S**, Borghi E, et al. Dysbiosis, host metabolism, and non-communicable diseases: trialogue in the inborn errors of metabolism. Front Physiol 2021;12:716520.
[25] Petrick JL, Florio AA, Koshiol J, et al. Prediagnostic concentrations of circulating bile acids and hepatocellular carcinoma risk: REVEAL-HBV and HCV studies. Int J Cancer 2020;147:2743–2753.
[26] Ceryak S, Bouscarel B, Malavolti M, et al. Extrahepatic deposition and cytotoxicity of lithocholic acid: studies in two hamster models of hepatic failure and in cultured human fibroblasts. Hepatology 1998;27:546–556.

Keywords: chronic hepatitis C (CHC); gut dysbiosis; *Blautia*; metabolomic analysis; transcriptional analysis (RNA-Seq).
Received 4 February 2025; received in revised form 15 June 2025; accepted 17 June 2025; Available online 24 June 2025

Journal of Hepatology, Volume *7*

Supplemental information

Restoration of the gut-microbiota-liver axis after hepatitis C virus eradication

Takako Inoue, Jiro Nakayama, Hiroshi Mori, Masaru Tanaka, Daisuke Nakagawa, Masaya Ohnishi, Yui Funatsu, Kei Moriya, Hideto Kawaratani, Hisayoshi Watanabe, Goki Suda, Yasuteru Kondo, Tatsuya Ide, Satoru Kakizaki, Satoshi Miuma, Atsushi Suetsugu, Kazuhito Kawata, Takao Watanabe, Etsuko Iio, Rie Momoda, Yutaka Suzuki, Akira Sakamaki, Tsunamasa Watanabe, Takehisa Watanabe, Katsuya Nagaoka, Yoichi Hiasa, Shuji Terai, Hitoshi Yoshiji, Atsushi Toyoda, Ken Kurokawa, and Yasuhito Tanaka

Restoration of the gut-microbiota-liver axis after hepatitis C virus eradication

Takako Inoue, Jiro Nakayama, Hiroshi Mori, Masaru Tanaka, Daisuke Nakagawa, Masaya Ohnishi, Yui Funatsu, Kei Moriya, Hideto Kawaratani, Hisayoshi Watanabe, Goki Suda, Yasuteru Kondo, Tatsuya Ide, Satoru Kakizaki, Satoshi Miuma, Atsushi Suetsugu, Kazuhito Kawata, Takao Watanabe, Etsuko Iio, Rie Momoda, Yutaka Suzuki, Akira Sakamaki, Tsunamasa Watanabe, Takehisa Watanabe, Katsuya Nagaoka, Yoichi Hiasa, Shuji Terai, Hitoshi Yoshiji, Atsushi Toyoda, Ken Kurokawa, Yasuhito Tanaka

Table of contents

Supplementary methods……………………….…………………………………2

Supplementary figures…………………...8

Supplementary tables...16

Supplementary references…………………………………..……..…………24

Supplementary methods

Patients enrolled into the study

The CHC patients (any HCV genotype) were enrolled at Nagoya City University Hospital (Aichi, Japan), Nara Medical University Hospital (Nara, Japan), Gifu University Hospital (Gifu, Japan), Kurume University Hospital (Fukuoka, Japan), Gunma University Hospital (Gunma, Japan), Nagasaki University Hospital (Nagasaki, Japan), Hamamatsu University Hospital (Shizuoka, Japan), Ehime University Hospital (Ehime, Japan), Niigata University Medical and Dental Hospital (Niigata, Japan), St. Marianna University Hospital (Kanagawa, Japan) from October 2013 to March 2022.

Enrolled patients had not taken ursodeoxycholic acid (UDCA) within the 6 months prior to sampling for this study. The inclusion and exclusion criteria, and the definition of each clinical stage in CHC, have been described in our previous papers [1, 2]. Fecal samples were used for gut microbiome analysis and the analysis of BA profiles, and the methods used are described below.

Healthy individuals enrolled for this study

The sampling from healthy individuals was performed as a part of the Asian Microbiome Project that investigates the basal microbiota of Japanese people. The inclusion and exclusion criteria have been described in our previous papers [1, 2].

Study design

This study was conducted retrospectively. Firstly, the cross-sectional study was performed to compare the fecal bacterial compositions of 272 subjects (174 CHC patients with HCV infection, 75 patients after SVR, and 23 healthy individuals). Of them, 166 CHC patients with HCV infection and 23 healthy individuals who had participated in our previous study [1] and their 16S rRNA sequence data were combined into this study. The details are shown in **Fig. 1 and Table S1**. In the cross-

sectional study, the patients who had achieved HCV eradication at least 24 weeks previously were defined as the patients after SVR. Regarding the patients with HCV infection, PNALT (n = 18) plus CH (n = 77) were defined together as the CH-HCV group (n = 95), and patients with LC (n = 51) and HCC (n = 28) were defined together as the LC/HCC-HCV group (n = 79). Regarding the patients after SVR, PNALT (n = 4) and CH (n = 25) were defined together as the CH-SVR group (n = 29), and patients with LC (n = 29) and HCC (n = 17) were defined together as the LC/HCC-SVR group (n = 46). We compared the gut microbiomes and BA composition of these four groups (CH-HCV, LC/HCC-HCV, CH-SVR, LC/HCC-SVR groups) and the healthy individuals.

Subsequently, 49 CHC patients enrolled in the cross-sectional study were also enrolled in the longitudinal study. In the longitudinal study, the gut microbiome during HCV infection was compared to that at 24 and 48 weeks after SVR. Samples obtained 48 weeks after SVR were provided by 29 of the 49 CHC patients. The number of subjects investigated in the longitudinal study satisfied the power of size; the minimum numbers of subjects were estimated to be 28 for the LC/HCC group and 15 for the CH group, by a power analysis using the cross-sectional data (**Fig. 4**) of mean and variance of abundance of genus *Blautia*, before and after SVR in LC/HCC and CH groups (GPower 3.1, a priori test in the Wilcoxon signed-rank matched-pairs test, a statistical power (1-β) of 0.8 and a type I (α) error rate of 0.05) [3]. The details of the subjects are shown in **Table S2**. The patients with PNALT (n = 4) and CH (n = 25) were defined together as the CH group (n = 29), and the patients with LC (n = 19) and HCC (n =1) were defined together as the LC/HCC group (n = 20) and we compared the gut microbiomes of these two groups.

The study design is shown as **Fig. 1,** and the characteristics of the CHC patients and healthy individuals are presented in **Table 1** (cross-sectional study) **and Tables S2-S3** (longitudinal study).

Gut microbiome analysis

Total bacterial DNA was isolated from stool samples using the bead-beating method, followed by phenol extraction [1]. The variable V1-V2 (27F-354R primers) or V3-V4 (341F-785R primers) regions of the 16S ribosomal RNA gene were amplified by polymerase chain reaction in the cross-sectional and longitudinal study, respectively, and then subjected to high-throughput sequencing using the MiSeq paired-end sequencing system (Illumina Inc., San Diego, CA, USA) [1]. The sequences obtained were processed using the QIIME2 platform (qiime2-2023.2, https://qiime2.org) [4]. Briefly, the paired-end sequences were merged, trimmed, denoised, and clustered into amplicon sequence variants (ASVs). The representative sequence of each ASV was taxonomically classified using the classify-sklearn program with the SILVA 138 database (https://www.arb-silva.de/). The statistics, ASV, and taxonomy tables in the 16S rRNA amplicon profiling are summarized in the **Tables S4-S8**.

For assessing the α-diversity of the gut microbiome in CHC patients during HCV infection, post-SVR at various clinical stages, and in healthy individuals, a rarefaction curve of the Shannon-Wiener index was constructed to reflect the ASV composition in each sample and increasing subsampling depth, in the QIIME2 platform. After the sampling depth was rarefied at 7,500 reads in each group, a significant difference of the Shannon-Wiener index in each group to the healthy group was examined by the nonparametric Dunnett's test in the R nparcomp package.

The β-diversity of gut microbiome was estimated using the Bray-Curtis and Jaccard distances, calculated based on the ASV composition of each sample. Biases in the alpha diversity between groups were calculated by the Permanova analysis in 'adonis' function in the R vegan package. The contribution of host factors to the ordination was calculated using the 'envfit' function in the R vegan package.

The genus composition of each sample was averaged within each group and examined by nonparametric Dunnett's test.

The statistical significance of the change in the longitudinal measure to compare the gut microbiome in CHC patients during HCV infection and after SVR was examined using the Wilcoxon signed-rank test in the Stata program (SE12.0). Correlation of the change in the abundance of each genus and ASV with the liver function parameters was examined by the generalized estimating equations (GEE) in the Stata program. In the GEE analysis, the relative abundance of each genus or ASV was normalized across all samples, each liver function parameter was converted to a Z-score, and applied for the GEE analysis with an unstructured correlation matrix and gaussian family speculation.

Measurement of the concentration of BAs

Of the 272 samples whose gut microbiota were analyzed, BA analysis was conducted on 176 patients whose sample volumes were sufficient for this purpose. The major 15 BAs in fecal samples from the 176 subjects (23 healthy individuals, 58 CH-HCV, 42 LC/HCC-HCV, 16 CH-SVR, 37 LC/HCC-SVR) were quantified using high-performance liquid chromatography-triple quadrupole mass spectrometry (Shimadzu, LCMS 8050) with nor-deoxycholic acid as an internal standard, as described previously [2]. The subjects included 100 CHC patients with HCV infection and 23 healthy individuals who were enrolled in our previous study [2] and their BA data were combined into this study. The relative abundance data of the 15 BAs in each sample was subjected to between-group statistical difference analysis, as well as the cluster analysis.

For cluster analysis, Euclidean distances between samples were calculated based on the BA composition data of 176 samples by the 'vegdist' function in the R vegan package. Based on the distance, the distribution of samples was displayed in the principal coordinate analysis (PCoA) and were then subjected to the pam clustering in the R cluster package, resulting in three clusters with the highest Calinski-Harabasz index and reasonable Silhouette width (0.47), calculated by the R clusterSim package.

The three clusters were defined by the most abundant BA, namely DCA, LCA, and UDCA types. The distribution of the three BA types was compared statistically between healthy individuals and CHC patient groups or between before and after SVR, among healthy individuals and CHC patients during HCV infection and after SVR at various clinical stages by Macnemar's test in the Stata program. The correlation of host factors to the BA-based ordination was analyzed by the envfit function in the R vegan package and displayed as vector arrows on the PCoA plot, in addition to the estimates of r^2 and probability "p".

To find genera statistically abundant or deficient in a certain BA type, the relative abundance of each genus in samples was compared by the pairwise Wilcoxon rank-sum test with adjustment by Benjamin-Hochberg procedure.

Analysis of whole-transcriptome sequencing (RNA-seq)

The procedure has been described in our previous paper [2]. We randomly enrolled 65 Japanese, biopsy-proven CHC patients with HCV infection, including mild CHC (F0–2, n = 22) and advanced CHC (F3–4, n = 43). Additionally, 28 CHC patients after SVR, including mild CHC (F0-2, n = 12) and advanced CHC (F3-4, n = 16), were enrolled in this study. The data were obtained from the International Cancer Genome Consortium (ICGC). Our HCC patients were also included in the project. For healthy liver controls, 12 patients with similar ages to the CHC patients were enrolled. These samples were surgical resections from organ donors. RNA-Seq data were collected from Sequence Read Archive. Statistical analysis was performed using the Kruskal-Wallis test. Subsequently, multiple comparisons were conducted using the Steel-Dwass test, followed by Bonferroni adjustment. Clinical and pathological data are summarized in **Table S9**.

Statistics

In all of the statistical analysis, a *p* value <0.05 was considered statistically significant. The specific statistical methods applied are detailed in the corresponding subjections. For selected analyses, post hoc power calculations were conducted using G*Power version 3.1.9.2 to obtain 1-β (power). For the GEE analysis, power (1-β) was manually calculated by using the estimated effect size (delta), residual standard deviation, and intra-subject correlation (rho), as GPower dose not support GEE models.

Study approval

Written informed consent was obtained from each individual and the study was approved by each institutional ethics committee, in accordance with the Declaration of Helsinki. The methods were carried out in accordance with the approved guidelines.

Accession numbers of pyrosequencing data

The raw sequence data have been deposited in the DNA Data Bank of Japan (DDBJ) sequence read archive (DRR584747-DRR584826 and DRR585283-DRR585554) under BioProject no. PRJDB18460 (PSUB023519) and PRJDB18519 (PSUB023615).

Supplementary figures

(A)

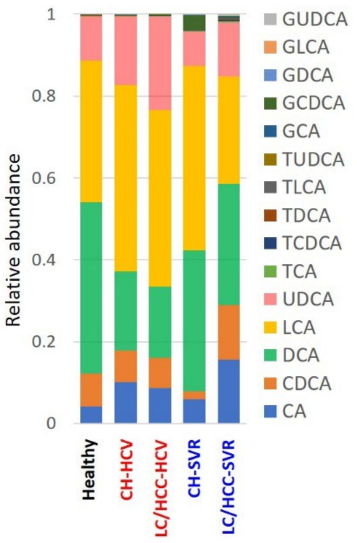

(B)

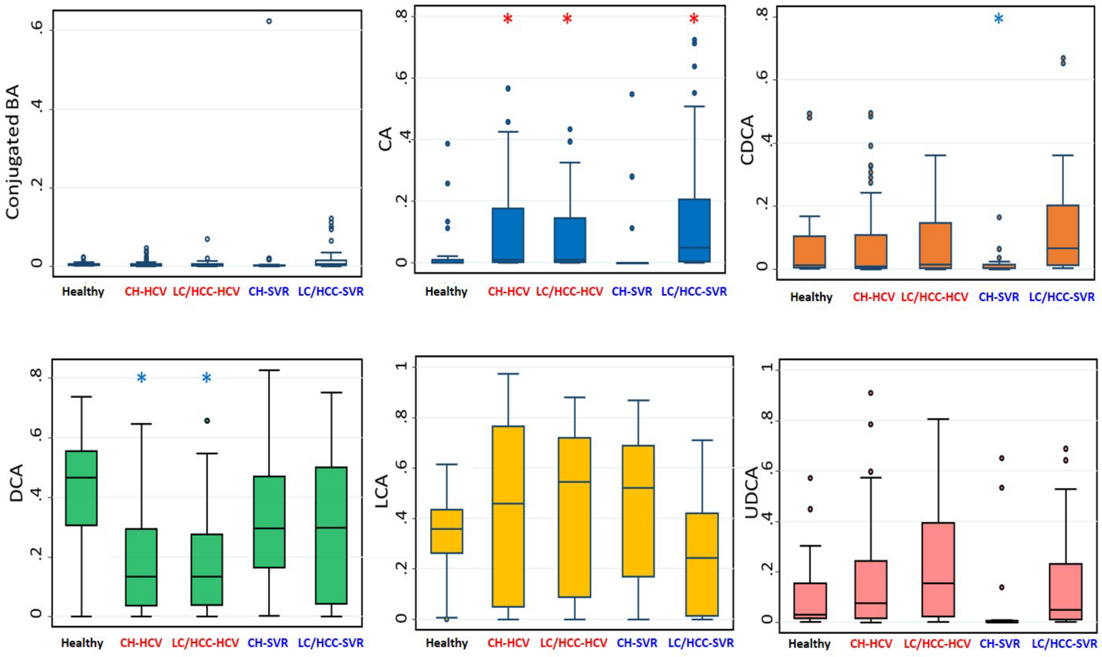

Fig. S1: Fecal BA profiles obtained from CHC patients and healthy individuals

(A) Stacked bar graph showing the relative BA composition in the fecal samples, averaged within each group. **(B)** Box plot showing the distribution of relative abundance of each BA in feces from each subject group. Red and blue stars above the

bars represent statistically significant higher or lower values than the healthy control group, respectively ($p < 0.05$ in Steel test).

Abbreviations: CHC, chronic hepatitis C; BA, bile acid; HCV, hepatitis C virus; CH, chronic hepatitis; LC, cirrhosis; HCC, hepatocellular carcinoma in cirrhosis; SVR, sustained virological response; CH-HCV group, persistently normal alanine aminotransferase (PNALT) or CH under HCV infection group; LC/HCC-HCV group, LC or HCC under HCV infection group; CH-SVR group, PNALT or CH after SVR group; LC/HCC-SVR group, LC or HCC after SVR group; healthy group, healthy individuals group; ASV, amplicon sequence variants; CA, cholic acid; CDCA, chenodeoxycholic acid; DCA, deoxycholic acid; LCA, lithocholic acid; UDCA, ursodeoxycholic acid; TCA, taurocholic acid; TCDCA, taurochenodeoxycholic acid; TDCA, taurodeoxycholic acid; TLCA, taurolithocholic acid; TUDCA, tauroursodeoxycholic acid; GCA, glycocholic acid; GCDCA, glycochenodeoxycholic acid; GDCA, glycodeoxycholic acid; GLCA, glycolithocholic acid; GUDCA, glycoursodeoxycholic acid.

(A)

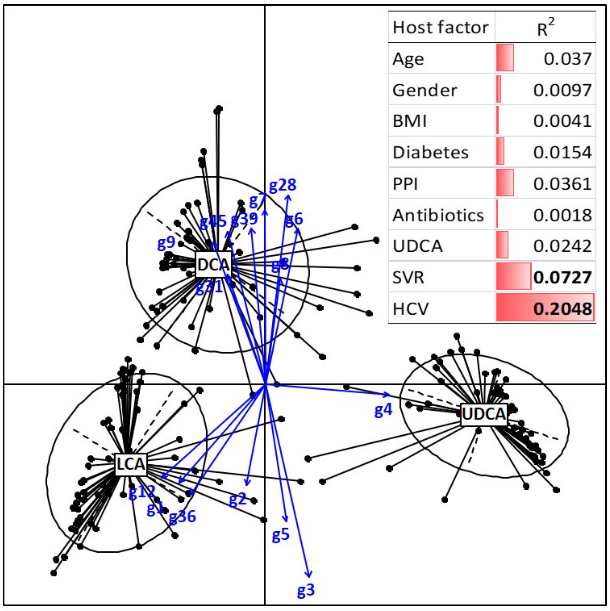

(B)

GeneID	Genus	CA	CDCA	DCA	LCA	UDCA
g1	*Bacteroides*	-0.085	-0.097	0.016	0.132	-0.028
g2	*Bifidobacterium*	-0.042	-0.110	-0.053	0.090	-0.055
g3	*Streptococcus*	0.114	0.027	**-0.250**	0.008	**0.149**
g4	*[Ruminococcus]_gnavus_group*	0.034	0.017	-0.057	0.035	-0.009
g5	*Anaerostipes*	-0.040	0.026	-0.038	-0.006	0.044
g6	*Prevotella_9*	0.001	0.010	0.086	-0.059	-0.108
g7	*Agathobacter*	-0.053	0.021	**0.205**	-0.046	-0.114
g8	*Blautia*	-0.005	0.082	0.087	-0.146	-0.003
g9	*Megamonas*	-0.045	-0.026	**0.168**	0.035	-0.101
g12	*Ligilactobacillus*	-0.042	-0.080	0.074	0.038	-0.114
g28	*Roseburia*	-0.053	0.020	0.144	-0.040	-0.081
g31	*Ruminococcus*	-0.012	0.001	0.039	-0.028	0.016
g36	*Escherichia-Shigella*	0.001	0.006	0.008	0.099	-0.029
g39	*Faecalibacterium*	-0.121	-0.037	**0.206**	-0.097	-0.064
g45	*Lachnoclostridium*	-0.091	-0.081	**0.239**	-0.002	**-0.165**

Fig. S2: Correlation of the abundance of each genus and host factors to the fecal BA ordination

(A) Correlation of the abundance of each genus and host factors to the principal coordination of subject' BA profile. The inlet table indicates the coefficient of determination (r) of each host factor to the BA principal coordination. Bold letters indicate the correlation to be significant ($p < 0.05$). Blue arrows indicate the correlation of each genus (gX, most 20 abundant genera listed in **(B)**) to the BA principal coordination ($p < 0.4$).

(B) Spearman correlation of the relative abundance of each genus to the relative abundance of each BA. Bold letters indicate the correlation with significance (p <0.05).

Abbreviations: BA, bile acid; PCA, principal component analysis; BMI, body mass index; PPI, proton pump inhibitor; UDCA, ursodeoxycholic acid; CHC, chronic hepatitis C; CH, chronic hepatitis; LC, cirrhosis; HCC, hepatocellular carcinoma in cirrhosis; SVR, sustained virological response; chenodeoxycholic acid; DCA, deoxycholic acid; LCA, lithocholic acid.

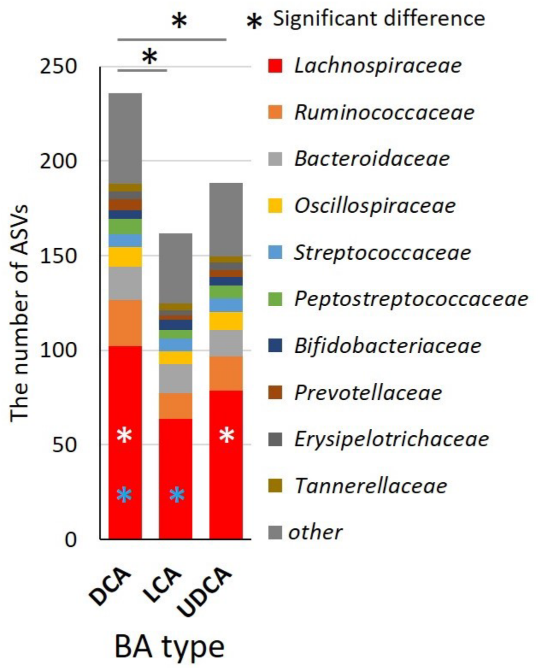

Fig. S3: Comparison of observed ASV numbers among different BA type groups
Stacked bar graph showing the number of ASVs observed in each BA type subject. Asterisks inside the bar indicate a significant difference in the number of ASVs, corresponding to family Lachnospiraceae, across 3 BA types. Asterisks over the bar indicate a significant difference of total number of ASVs observed in each BA type. The data were averaged within each BA type.

Abbreviations: ASV, amplicon sequence variant; BA, bile acid; chenodeoxycholic acid; DCA, deoxycholic acid; LCA, lithocholic acid; UDCA, ursodeoxycholic acid.

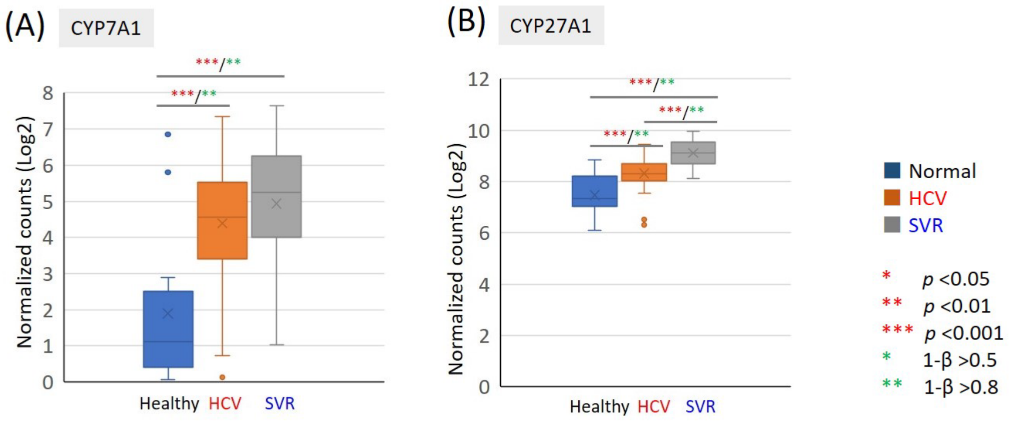

Fig. S4. Changes in the gene expression levels of enzymes involved in BA biosynthesis, in the liver after SVR

Expression in the liver of genes involved in the metabolism of BAs in 65 CHC patients (F0-2 [n = 22] and F3-4 [n = 43]) and individuals with healthy livers (n = 12) by RNA-Seq. Statistical analysis was performed by Kruskal-Wallis multiple comparison. *P*-values were adjusted using the Benjamini-Hochberg method. Red and green asterisks show the significant differences between the groups and the statistical power, respectively. (**A**) CYP7A1, (**B**) CYP27A1.

Abbreviations: BA, bile acid; SVR, sustained virological response; CHC, chronic hepatitis C; RNA-Seq, transcriptional analysis; Healthy, individuals with healthy liver; CYP7A1, cholesterol 7α-hydroxylase; CYP27A1, sterol 27-hydroxylase.

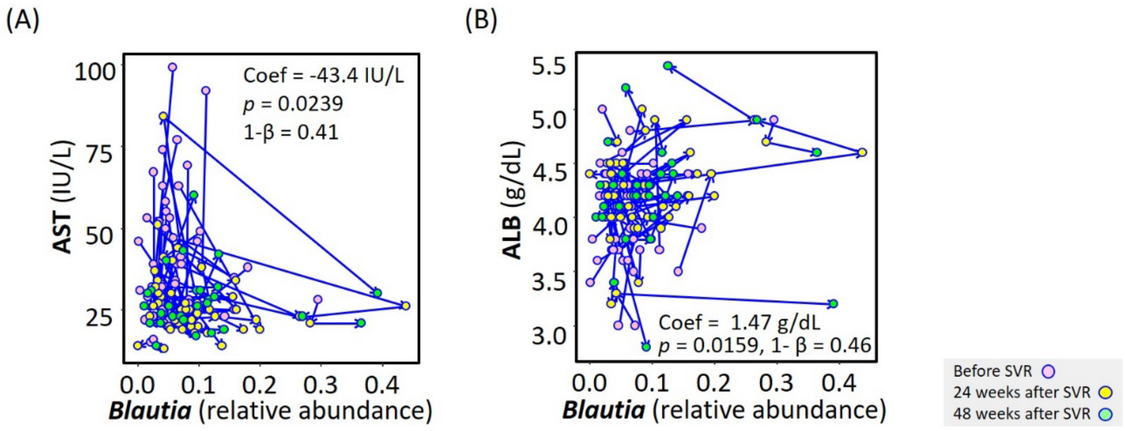

Fig. S5. Longitudinal changes of the gut microbiome after SVR and their association with liver inflammation indicators

Longitudinal changes in the abundance of *Blautia*, (**A**) AST and (**B**) ALB. Pink, yellow, and green dots represent data during HCV infection, SVR24 and SVR48, respectively. Arrow lines connect data from the same individuals over time.

Abbreviations: SVR, sustained virological response; ALT, alanine-2-oxoglutarate aminotransferase; AST, asparate-2-oxoglutarate aminotransferase; ALB, serum albumin; HCV, hepatitis C virus; SVR24, the status of 24 weeks after SVR; SVR48, the status of 48 weeks after SVR.

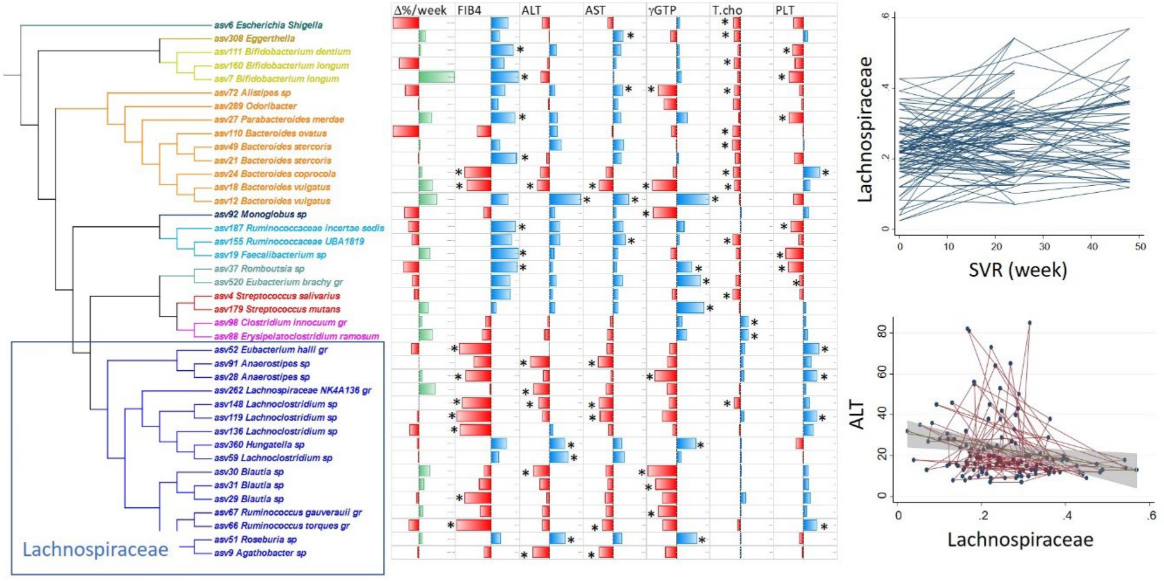

Fig. S6. Correlation with the abundance of each ASV to the liver function

The correlation between the change in abundance of each ASV and the alteration in liver function indicators before and after HCV elimination is shown in the bar graph besides the phylogenetic tree of each ASV. In the horizontal bar graphs, the column to the right of each ASV name illustrates the occupancy percentage change of each ASV, before and after achieving SVR. Green indicates an increase and red a decrease in ASVs (%). The subsequent columns to the right demonstrate the relationship between liver function indicators and each ASV. Red indicates an inverse correlation, while blue signifies a direct correlation. The ASVs named in dark blue letters belong to the order *Lachnospirales.* Statistically significant correlations are indicated by asterisks (in the GEE analysis, *p <0.05). The upper right line graph indicates the change in the total abundance of ASV belonging to order *Lachnospirales* over time post-SVR. The lower right line graph shows the correlation between the change in the ALT levels and change in the abundance of *Lachnospirales.* Each dot represents ALT and the abundance of *Lachnospirales* in each sample.

Abbreviations: SVR, sustained virological response; FIB4, fibrosis-4 index; ALT, alanine-2-oxoglutarate aminotransferase; AST, asparate-2-oxoglutarate aminotransferase; γGTP, γ-glutamyl transpeptidase; t-cho, total cholesterol; PLT, platelet count.

Supplementary tables

<table>
<tr><th colspan="2">Patients profile</th><th>Previous report (n = 166) [1]</th><th colspan="2">This study (n = 249)</th></tr>
<tr><td rowspan="5">Under HCV infection</td><td>PNALT</td><td>18</td><td>18</td><td rowspan="2">CH-HCV group (n = 95)</td></tr>
<tr><td>CH</td><td>84</td><td>77</td></tr>
<tr><td>LC</td><td>40</td><td>51</td><td rowspan="2">LC/HCC-HCV group (n = 79)</td></tr>
<tr><td>HCC</td><td>24</td><td>28</td></tr>
<tr><td>Total</td><td>166</td><td colspan="2">174</td></tr>
<tr><td rowspan="5">After SVR</td><td>PNALT</td><td rowspan="5"></td><td>4</td><td rowspan="2">CH-SVR group (n = 29)</td></tr>
<tr><td>CH</td><td>25</td></tr>
<tr><td>LC</td><td>29</td><td rowspan="2">LC/HCC-SVR group (n = 46)</td></tr>
<tr><td>HCC</td><td>17</td></tr>
<tr><td>Total</td><td colspan="2">75</td></tr>
</table>

Table S1: CHC patients enrolled in cross-sectional study

Abbreviations: CHC, chronic hepatitis C; HCV, hepatitis C virus; CH, chronic hepatitis; LC, cirrhosis; HCC, hepatocellular carcinoma in cirrhosis; SVR, sustained virological response; CH-HCV group, persistently normal alanine aminotransferase (PNALT) or CH under HCV infection group; LC/HCC-HCV group, LC or HCC under HCV infection group; CH-SVR group, PNALT or CH after SVR group; LC/HCC-SVR group, LC or HCC after SVR group; healthy group, healthy individuals group.

Enrolled patients		Numbers of provided samples		
		Under HCV infection	24 weeks after SVR	48 weeks after SVR
PNALT (n = 4)	CH group (n = 29)	29	29	18
CH (n = 25)				
LC (n =19)	LC/HCC group (n = 20)	20	20	11
HCC (n = 1)				
Total	49	49	49	29

Table S2: CHC patients enrolled in longitudinal study

Abbreviations: CHC, chronic hepatitis C; HCV, hepatitis C virus; CH, chronic hepatitis; LC, cirrhosis; HCC, hepatocellular carcinoma in cirrhosis; SVR, sustained virological response; CH-HCV group, persistently normal alanine aminotransferase (PNALT) or CH under HCV infection group; LC/HCC-HCV group, LC or HCC under HCV infection group; CH-SVR group, PNALT or CH after SVR group; LC/HCC-SVR group, LC or HCC after SVR group; healthy group, healthy individuals group.

Characteristics	Category (Number of candidates)		*p* values
	CH group (n = 29)	LC/HCC group (n = 20)	
Gender (M/F）	9/20	7/13	0.77
Age (years)	66.1 ± 12.6	72.7 ± 5.9	0.057
PLT (× 104/mm3)	20.4 ± 5.7	11.8 ± 5.7	< 0.0001
PT (%)	94.2 ± 9.5	84.8 ± 14.8	0.012
Alb (g/dL)	4.1 ± 0.4	4.0 ± 0.4	0.2
AST (IU/L)	37.3 ± 22.3	44.9 ± 13.0	0.18
ALT (IU/L)	30.1 ± 20.4	32.3 ± 11.9	0.68
γGT (IU/L)	22.3 ± 10.3	56.1 ± 57.5	0.0032
T-Bil (mg/dL)	1.1 ± 1.2	0.9 ± 0.4	0.58
AFP (ng/ml)	4.8 ± 7.2	11 ± 8.9	0.01
PIVKA-II (mAU/ml)	20.7 ± 13.6	23.2 ± 11	0.5
FIB-4 index	2.4 ± 1.2	6.3 ± 3.6	< 0.0001

Table S3: Demographics and clinical characteristics of CHC patients (n = 49) for longitudinal study

Continuous data are expressed as means ± standard deviation. Bold letters indicate a significant difference in one-way ANOVA followed by Tukey–Kramer post analysis.

Abbreviations: CHC, chronic hepatitis C; CH, persistently normal alanine aminotransferase or chronic hepatitis; LC/HCC, cirrhosis or hepatocellular carcinoma; healthy, healthy individuals; M, male; F, female; PLT, platelet count; PT, prothrombin time; Alb, serum albumin; AST, asparate-2-oxoglutarate aminotransferase; ALT, alanine-2-oxoglutarate aminotransferase; γGT, γ-glutamyl transpeptidase; T-Bil, total bilirubin; AFP, alpha fetoprotein; PIVKA-II, protein induced by vitamin K absence or antagonist-II; FIB-4 index, fibrosis-4 index.

**Cross-sectional Study (n = 272)**	input paired seqs.	quality pass merged seqs.	ASV	Genus	Family	Phylum
total	14,548,385	10,751,608	12,424	331	104	13
mean per sample	53,487	39,528	180.5	54.0	25.1	5.4
std per sample	52,455	40,981	126.8	23.7	7.5	1.1
max per sample	245,711	190,575	714	132	49	9
min per sample	11,674	7,740	19	12	10	3

**Longitudinal Study (n = 149)**	input paired seqs.	quality pass merged seqs.	ASV	Genus	Family	Phylum
total	15,272,826	6,936,502	3,846	333	95	15
mean per sample	120,258	54,618	149.6	66.65354	28.92913	5.9
std per sample	35,878	19,231	59.6	24.79451	8.14178	1.3
max per sample	234,529	107,499	331	127	53	10
min per sample	32,228	4,795	48	21	13	3

Table S4: Statistics in 16S rRNA profiling in this study

Abbreviations: ASV, amplicon sequence variants.

Table S5: ASV table in the cross-sectional study

Table S6: Taxonomy table in the cross-sectional study

Table S7: Taxonomy table in the longitudinal study

Table S8: Taxonomy table in the longitudinal study

Due to the large size of **Tables S5-S8**, they are provided separately as an Excel file. Please refer to the attached file for detailed data.

Characteristics at the time of liver biopsy	Category (Number of candidates)		
	CHC under HCV infection (n = 65)	CHC after SVR (n = 28)	Healthy (n = 12)
Gender (M/F)	44/21		9/3
Age (years)	$71.8 \pm 7.0^{a, b}$	$65.4 \pm 6.3^{a, c}$	$54.6 \pm 14.0^{b, c}$
Fibrosis stage (F0/F1/F2/F3/F4)	2/8/12/12/31	0/6/6/8/8	n.d.

Table S9: Characteristics of CHC patients (n = 64) and individuals with healthy livers (n = 12) whose liver tissues were used for RNA-Seq

Age data are expressed as mean ± standard deviation. Superscript letters indicate a significant difference in one-way ANOVA followed by Tukey-Kramer post-hoc analysis (p <0.0001: a, b and c).

Abbreviations: CHC, chronic hepatitis C; RNA-Seq, transcriptional analysis; HCV, hepatitis C virus; SVR, sustained virological response; Healthy, individuals with healthy liver; M, male; F, female; n.d., not determined.

	Dim1	Dim2	r2	Pr(>r)	
Age	0.08518	0.99637	0.0074	0.411	
Gender	0.79319	0.60897	0.0156	0.178	
BMI	-0.6382	0.76987	0.0017	0.846	
Diabetes	0.69724	0.71684	0.0124	0.24	
PPI	0.58862	0.80841	0.0534	0.003	**
Antibiotics	-0.1046	0.99451	0.0024	0.755	
H2.blocker	0.67999	0.73322	0.0256	0.044	*
UDCA	0.91945	-0.3932	0.0189	0.131	
Beta.blocker	-0.59632	-0.80275	0.0133	0.213	
SVR	-0.56379	-0.82592	0.5976	0.001	***
ClinicalStage			0.3091	0.001	***
CH-HCV	0.0622	0.0577	0.3091		
LC/HCC-HCV	0.0302	0.069	0.3091		
CH-SVR	-0.0997	-0.1557	0.3091		
LC/HCC-SVR	-0.0971	-0.0788	0.3091		

Table S10: Correlation of hosts and possible confounding factors with the PCoA ordination based on Bray-curtis distance across ASV composition of the 272 samples

Abbreviations: PCoA, principal coordinate analysis, ASV, amplicon sequence variants; Dim, Dimension; BMI, body mass index; PPI, proton pump inhibitor; H2 blocker, histamine H2-receptor; UDCA, ursodeoxycholic acid; SVR, sustained virological response; CH-HCV, persistently normal alanine aminotransferase (PNALT) or CH under HCV infection group; LC/HCC-HCV, LC or HCC under HCV infection group; CH-SVR, PNALT or CH after SVR group; LC/HCC-SVR, LC or HCC after SVR group.

	Dim1	Dim2	r2	Pr(>r)	
Age	0.07387	0.99727	0.0074	0.419	
Gender	0.7165	0.69759	0.0141	0.191	
BMI	-0.7349	0.67818	0.0017	0.833	
Diabetes	0.63166	0.77524	0.0121	0.23	
PPI	0.54035	0.84144	0.0524	0.005	**
Antibiotics	-0.19762	0.98028	0.0028	0.724	
H2.blocker	0.61152	0.79123	0.0251	0.055	.
UDCA	0.95311	-0.30264	0.0189	0.072	.
Beta.blocker	-0.47819	-0.87826	0.0147	0.185	
SVR	-0.47747	-0.87865	0.5933	0.001	***
ClinicalStage			0.3004	0.001	***
CH-HCV	0.0438	0.05			
LC/HCC-HCV	0.0194	0.056			
CH-SVR	-0.0655	-0.128			
LC/HCC-SVR	-0.0687	-0.0691			

Table S11: Correlation of hosts and possible confounding factors with the PCoA ordination based on Jaccard distance across ASV composition of the 272 samples

Abbreviations: PCoA, principal coordinate analysis, ASV, amplicon sequence variants; Dim, dimension; BMI, body mass index; PPI, proton pump inhibitor; H2 blocker, histamine H2-receptor; UDCA, ursodeoxycholic acid; SVR, sustained virological response; CH-HCV, persistently normal alanine aminotransferase (PNALT) or CH under HCV infection group; LC/HCC-HCV, LC or HCC under HCV infection group; CH-SVR, PNALT or CH after SVR group; LC/HCC-SVR, LC or HCC after SVR group.

Supplementary references

[1] Inoue T, Nakayama J, Moriya K, et al. Gut Dysbiosis Associated With Hepatitis C Virus Infection. Clin Infect Dis 2018;67:869-877.

[2] Inoue T, Funatsu Y, Ohnishi M, et al. Bile acid dysmetabolism in the gut-microbiota-liver axis under hepatitis C virus infection. Liver Int 2022;42:124-134.

[3] Faul F, Erdfelder E, Buchner A, et al. Statistical power analyses using G*Power 3.1: tests for correlation and regression analyses. Behav Res Methods 2009;41:1149-1160.

[4] Bolyen E, Rideout JR, Dillon MR, et al. Reproducible, interactive, scalable and extensible microbiome data science using QIIME 2. Nat Biotechnol 2019;37:852-857.
